# Exploration of Q-Marker of Rhubarb Based on Intelligent Data Processing Techniques and the AUC Pooled Method

**DOI:** 10.3389/fphar.2022.865066

**Published:** 2022-03-21

**Authors:** Jiayun Chen, Xiaojuan Jiang, Chunyan Zhu, Lu Yang, Minting Liu, Mingshe Zhu, Caisheng Wu

**Affiliations:** ^1^ Fujian Provincial Key Laboratory of Innovative Drug Target Research and State Key Laboratory of Cellular Stress Biology, School of Pharmaceutical Sciences, Xiamen University, Xiamen, China; ^2^ College of Pharmacy, Jiamusi University, Jiamusi, China; ^3^ MassDefect Technologies, Princeton, NJ, United States

**Keywords:** rhubarb, Q-marker, AUC-pooled method, intelligent data processing techniques, rhein

## Abstract

Rhubarb, as a traditional Chinese medicine, has several positive therapeutic effects, such as purging and attacking accumulation, clearing heat and purging fire, cooling blood, and detoxification. Recently, Rhubarb has been used in prescriptions for the prevention and treatment of COVID-19, with good efficacy. However, the exploration of effective quantitative approach to ensure the consistency of rhubarb’s therapeutic efficacy remains a challenge. In this case, this study aims to use non-targeted and targeted data mining technologies for its exploration and has comprehensively identified 72 rhubarb-related components in human plasma for the first time. In details, the area under the time-concentration curve (AUC)-pooled method was used to quickly screen the components with high exposure, and the main components were analyzed using Pearson correlation and other statistical analyses. Interestingly, the prototype component (rhein) with high exposure could be selected out as a Q-marker, which could also reflect the metabolic status changes of rhubarb anthraquinone in human. Furthermore, after comparing the metabolism of different species, mice were selected as model animals to verify the pharmacodynamics of rhein. The *in vivo* experimental results showed that rhein has a positive therapeutic effect on pneumonia, significantly reducing the concentration of pro-inflammatory factors [interleukin (IL)-6 and IL-1β] and improving lung disease. In short, based on the perspective of human exposure, this study comprehensively used intelligent data post-processing technologies and the AUC-pooled method to establish that rhein can be chosen as a Q-marker for rhubarb, whose content needs to be monitored individually.

## Introduction

Traditional Chinese medicine (TCM) is a traditional treasure of China, with thousands of years of clinical experience. It plays an important role in disease prevention and treatment, escorting human health. However, due to the origin of varieties, growth conditions, processing process, storage conditions and other reasons, the types and contents of TCM ingredients might be easily changed, which has a significant impact on safety and effectiveness (Bai et al., 2018; Zhang et al., 2018; Ren et al., 2020). Therefore, it is necessary to establish the perfect quality standard for TCM to ensure the consistency of efficacy. For this purpose, Liu *et al.* proposed the concept of Quality-marker (Q-marker) to provide a novel research direction for the quality evaluation of TCM (Liu et al., 2016). Specifically, Q-marker is a component inherent in TCM or generated in the preparation process, which can be qualitatively and quantitatively analyzed. More importantly, it should also have good biological activity, with clear therapeutic effects *in vivo*. However, the exploration to screen out a suitable Q-marker from hundreds or thousands of components of TCM remains a challenge.

As a typical case, Rhubarb is a traditional Chinese medicine (TCM) commonly used clinically, which is mainly from the dried roots and rhizomes of *Rheum palmatum* L., *Rheum tangicum* Maxim. ex Balf., or *Rheum officinale* Baill. Rhubarb has the following effects when used clinically: purging and attacking accumulation, clearing away heat and fire, cooling blood and detoxification, removing blood stasis and clearing menstruation, removing dampness, and reducing yellowness (Chinese Pharmacopoeia Commission, 2020). At the same time, as a part of the “three-medicines and three-decoctions” Huashi Baidu decoction and Lianhua Qingwen capsules, rhubarb has also played an irreplaceable role in the treatment of new coronary pneumonia (COVID-19), showing potential anti-inflammatory effect (Huang et al., 2020; Zhuang et al., 2020). In order to guarantee the efficacy of rhubarb, it is necessary to conduct quality control on it. Based on different preparation methods, the 2020 Chinese Pharmacopoeia calculates the content of total anthraquinone and free anthraquinone (above 1.5 and 0.02%, respectively) as to control the quality of rhubarb, in terms of the total amount of aloe-emodin, rhein, emodin, chrysophanol, and physcion. It is generally believed that substances that produce curative effects are TCM prototype components or their metabolites, which enter systemic circulation to reach the target organs (Li et al., 2018; Zhou et al., 2020; Zhu et al., 2020; Yang et al., 2021). Although anthraquinones show good pharmacological activities *in vitro*, including laxative, anticancer, hepatoprotective, anti-inflammatory, antibacterial, analgesic, and other effects (Xiang et al., 2020). However, they might not be absorbed into the body and exhibit disappointing effects *in vivo*, which might lead to an undesired failure in quality control (He et al., 2018). Therefore, it is necessary to pay attention to the pharmacokinetics of TCM *in vivo*. Carrying out research from the *in vivo* perspective will be more conducive to discover the pharmacodynamic material basis of rhubarb and determine the most suitable Q-marker.

The components of rhubarb are complex. It is very difficult to carry out pharmacokinetic studies on multiple components simultaneously. The area under the time-concentration curve (AUC) pooled method can simplify the traditional pharmacokinetic experiment model by using the same principle (Hop et al., 1998). The plasma volume ratio at each time point can be obtained using the following formula $v_{1}∶v_{2}∶\cdots ∶v_{i}=\left(t_{1}-t_{0}\right)∶\left(t_{2}-t_{0}\right)∶\left(t_{3}-t_{1}\right)∶\cdots ∶\left(t_{i}-t_{i-1}\right)$ . The plasma is then mixed. In a single plasma sample after mixing, the measured concentration multiplied by the time (tm) of the last blood collection is theoretically equal to AUC_0-tm_. Compared with the traditional method, the AUC pooled method greatly reduces the number of samples and the analysis time, allowing experimental results to be obtained more quickly and easily. This method is especially suitable for the study of metabolism *in vivo*. It is worth mentioning that there are metabolic differences in the effects of TCM among different species due to differences in the subtypes and activities of metabolic enzymes (Martignoni et al., 2006; Li et al., 2020; Hammer et al., 2021). Therefore, using the AUC pooled method can also quickly obtain the exposure proportions of the prototype and metabolite components in different species. Thus, species that are similar to humans are used as models for drug efficacy verification.

Based on ultra-performance liquid chromatography-high resolution mass spectrometry (UPLC-HRMS) technology and various intelligent mass spectrometry data processing technologies that were developed previously (Zhu et al., 2020), this study aims to conduct the pioneer comprehensive exploration of rhubarb-related components in humans. Due to the safety consideration in human trials, only marketed drug, which contains rhubarb as main part of formulation and has been approved by National Medical Products Administration, can be suitable for this study. In this case, Jiuzhi Dahuang Wan (JZDHW) has met the above requirement and been chosen as the typical medicine formulae in this exploration. The formulae instructions show that the ingredients are rhubarb, while the auxiliary material is rice wine. Due to the simple compositions of JZDHW, impacts of other components on this metabolic study can be effectively avoided, which further facilitates the discovery of the actual substances in rhubarb exposed to humans. More importantly, combined with the AUC pooled method, this study could visually display the prototype and metabolite components with high exposure. Furthermore, the relationship of exposure between different prototype and metabolite components could be obtained through correlation statistical analysis. After that, the Q-markers of rhubarb were rapidly screened from the perspective of human exposure, and the Q-markers were pharmacodynamically verified.

## Materials and Methods

### Chemicals and Reagents

JZDHW (batch number: 0470049) was purchased from Tianjin lerentang pharmaceutical factory (Tianjin, China). Chrysazin, *p*-coumaric acid, gallic acid (*E*)-piceatannol, aloe-emodin, emodin, chrysophanol, physcion, rhein, kaempferol, catechin, quercetin, rutin, lindleyin, isoquercitrin, sennoside C, emodin 8-*O*-*β-d*-glucoside, rhapontin, rhein 8-*O*-*β-d*-glucoside, quercitrin, and sennoside A were supplied by Baoji Chenguang Biotechnology Co., Ltd. (Baoji, China). Sprague Dawley (SD) rat blank plasma and human blank plasma were provided by Shanghai Yuanye Bio-Technology Co., Ltd. (Shanghai, China). Oasis^®^ HLB 6 cc (500 mg) extraction cartridges were purchased from Waters Corporation (Milford, MA, United States). Methanol and acetonitrile were purchased from TEDIA (Fairfield, OH, United States). Formic acid was purchased from Anaqua Chemical Supply (Wilmington, DE, United States). Ultra-pure water was prepared using a Milli-Q water purification system (Bedford, MA, United States). Heparin sodium was purchased from Shanghai Aladdin Bio-Chem Technology Co., LTD. (Shanghai, China). LPS was purchased from Beijing Solarbio Technology Co., LTD. (Beijing, China). Mouse interleukin (IL)-6 uncoated ELISA, mouse tumour necrosis factor alpha (TNFα) uncoated ELISA, and mouse IL-1β uncoated ELISA kits were obtained from Thermo Fisher Scientific Co. (Waltham, MA, United States). Carboxymethyl cellulose (CMC) was purchased Shanghai Macklin Biochemical Co., Ltd. (Shanghai, China).

### JZDHW Composition Analysis

JZDHW powder (20 mg) was dissolved in 1 ml 70% methanol, sonicated, vortexed, and filtered using a 0.22 μm filter membrane to obtain a final test solution.

### Plasma Sample Collection

#### Human Plasma Sample Collection

After 12 h of fasting, three healthy female subjects (authors of this study) were orally administered one bag of JZDHW (6 g). Intravenous blood sampling (1% heparin sodium anticoagulant) was conducted at a total of 12 time points (before administration and 0.25, 0.5, 1, 2, 3, 4, 6, 8, 10, 12, and 24 h after administration). The blood samples were then centrifuged for 10 min at 4,000 rpm/min, and the supernatants were stored in aliquots at −80°C. Subject’s informed consent were obtained and the work described was carried out in accordance with The Code of Ethics of the World Medical Association (Declaration of Helsinki).

#### Animal Plasma Sample Collection

A total of 12 female SD rats (180–220 g) and 56 female C57BL/6 mice (18–22 g, except for eight mice at 12 and 24 h, four mice at other time points, respectively) were purchased from Shanghai SLAC Laboratory Animal Co., Ltd. (Shanghai, China). After 1 week of adaptive feeding, followed by fasting for 12 h, rhubarb solution (JZDHW were ground to a powder and dissolved in ultra-pure water) was administered by oral gavage at a dose of 8 g/kg. Blood samples (1% heparin sodium anticoagulant) were collected before administration and 0.25, 0.5, 1, 2, 3, 4, 6, 8, 10, 12, and 24 h after administration, after which all samples were centrifuged at 4,000 rpm/min for 10 min. The supernatant was then aliquoted and stored at −80°C. All animal care and experimental procedures in this experiment conform to the Guide for the Care and Use of Laboratory Animals of Xiamen University (the ethic approval number: XMULAC20210101).

### Sample Preparation

#### Pre-Treatment for the Mixed Plasma-AUC Pooled Method

A total of 2.5, 5, 7.5, 15, 20, 20, 30, 40, 40, 40, 140, and 120 μL of human plasma was collected at 0, 0.25, 0.5, 1, 2, 3, 4, 6, 8, 10, 12, and 24 h after administration, respectively. The human plasma was then mixed uniformly to obtain 480 μL plasma, followed by addition of 20 μL of internal standard (50 μg/ml chrysazin, Protein was then precipitated with three times the volume of methanol. The supernatant after centrifugation was blown dry with nitrogen, and the residue was reconstituted with 100 μL of 70% methanol for testing. Each set of samples was evaluated in triplicate. The rat/mouse plasma samples were evaluated using the same procedure as that for human plasma.

Protocols for the solid-phase extraction processing method, AUC pooled method development, and verification of quality control sample preparation and pre-processing methods are provided in the Supplementary Materials. Additionally, the reasons for using chrysazin as the internal standard are as follows. Firstly, chrysazin is an anthraquinone compound, whose physicochemical properties are similar to those of the main components of rhubarb. Secondly, chrysazin and its isomers are neither contained in rhubarb itself nor in plasma samples. Last but not least, chrysazin does not chemically react with the tested sample, and its chromatographic peak can be completely separated.

#### Pre-Treatment Method for Human Plasma Samples at a Single Time Point

480 μL human plasma was collected at each of 12 time points, followed by addition of 20 μL of internal standard (50 μg/ml chrysazin). Protein was precipitated with three times the volume of methanol. The supernatant after centrifugation was blown dry with nitrogen, and the residue was reconstituted with 100 μL of 70% methanol for testing. Each set of samples was evaluated in triplicate.

### UPLC-HRMS Analysis

The samples were analysed using a Thermo Fisher Q Exactive Orbitrap liquid chromatography with tandem mass spectrometry system equipped with electrospray ionization (Thermo Fisher Scientific) in negative ion mode, which was controlled by Thermo Xcalibur 3.0.63 (Thermo Fisher Scientific). An ACQUITY UPLC CSH C18 column (50 mm × 2.1 mm, 1.7 μm; Waters Corporation) was used to separate the sample at a temperature of 35°C. Mobile phase A was H_2_O with 0.1% formic acid, and mobile phase B was 100% acetonitrile with a flow rate of 0.3 ml/min. The injection volume was set at 3 μL. The mobile phase gradient was set as follows: 0–7 min, 95%–60% (A); 7–15.5 min, 60%–40% (A); 15.5–18 min, 40%–20% (A); 18–20 min, 20%–5% (A); 20–23 min, 5%–5% (A); 23–23.1 min, 5%–95% (A); and 23.1–27 min, 95%–95% (A).

The MS parameters were set as follows: full MS resolution 35,000, scanning range *m/z* 100–1,000, dd-MS2 resolution 17,500, collision energy 35%, spray voltage 3.5 kV, capillary temperature 320°C, sheath gas (N2) flow rate 35 arb, auxiliary gas (N2) flow rate 10 arb, and sweep gas (N2) flow rate five arb.

### Pharmacodynamic Verification of the Pneumonia Model

A total of 40 female C57BL/6 mice, 18–22 g, were purchased from Shanghai SLAC Laboratory Animal Co., Ltd. The mice were randomly divided into five groups: the control group (A), model group (B), rhein group (C), and JZDHW group (D). The mice were reared adaptively for 1 week. Before modelling experiments, groups A and B were administered with 0.4% CMC, group C was administered with 85 mg/kg rhein, and group D was administered with 1.6 g/kg JZDHW at a volume of 20 ml/kg. All treatments were administered by oral gavage. Immediately following pre-treatment, a pneumonia model was established using the LPS nasal drip method. Mice were anesthetized by intraperitoneal injection of chloral hydrate. Treatments were orally administered every 12 h, followed by six consecutive administrations. The mice were sacrificed 12 h after the last administration, followed by collection of the serum and lung tissue. ELISA kits were used to detect the changes in serum inflammatory factors (TNFα, IL-6, and IL-1β). GraphPad Prism 8.0.2 software (GraphPad Software, San Diego, CA, United States) was used for statistical analysis. The significance of the data was evaluated using a two-tailed Student’s t-test and one-way analysis of variance. Significant differences between groups are represented by * for *p* < 0.05, ** for *p* < 0.01, and *** for *p* < 0.001. Haematoxylin and eosin staining was used to analyse the pathological condition of lung tissue. In addition, we established a pancreatitis model to supplement the anti-inflammatory activity of JZDHW components. For detailed experimental methods regarding the pancreatitis model, please see the Supplementary Materials.

## Results

### Analysis of JZDHW Components

**FIGURE 1:**
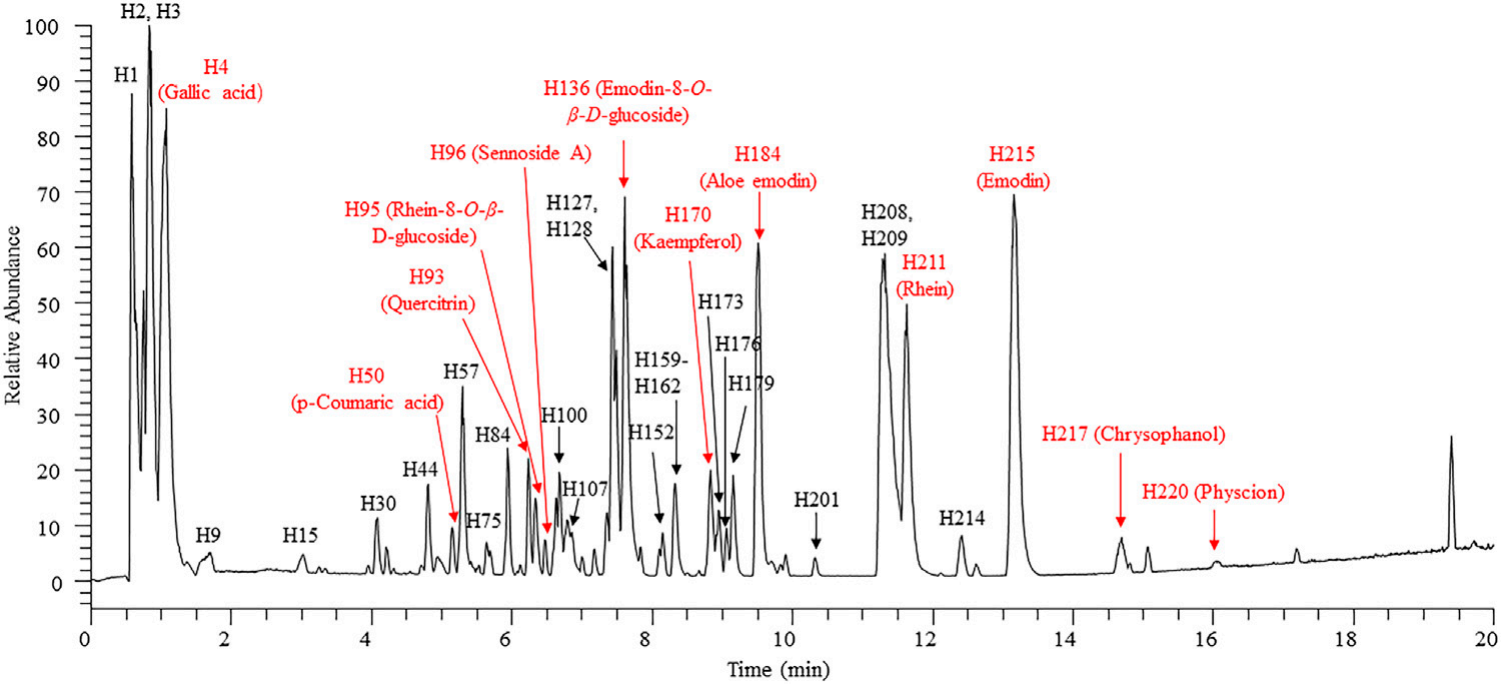
High-resolution total ion chromatogram of rhubarb. Red: compounds verified by reference substances.

The compositions of rhubarb anthraquinones are relatively similar in structure. To achieve the most effective separation of the compounds, we utilized an ACQUITY UPLC CSH C18 (50 mm × 2.1 mm, 1.7 μm) column and optimized the elution conditions. We selected three typical rhubarb anthraquinone reference substances (rhein, emodin, and chrysophanol; 10 μg/ml) to optimize the MS parameters, including the appropriate ionization mode (positive ion mode/negative ion mode), spray voltage, capillary temperature, capillary voltage, RF lens, flow rate of auxiliary gas, and other parameters. According to the final optimized conditions, JZDHW test solution was analysed by UPLC-HRMS. The total ion chromatogram is shown in Figure 1, with the corresponding data shown in Supplementary Table S1. A total of 223 compounds were detected from JZDHW. Compound identification was completed using Compound Discoverer 3.1 software (Thermo Fisher Scientific) combined with data reported in the literature (Lin et al., 2006; Cao et al., 2017; Niziol et al., 2017; Xian et al., 2017; Qin et al., 2020). The main parameters of the software were set as follows: Align Retention Times: Maximum Shift: 2 min; Mass tolerance: 5 ppm; Detect Compound: Mass Tolerance: 5 ppm; Intensity Tolerance: 30%; Min. Peak Intensity: 500,000; Data Sources: mzCloud, mzVault, MassList, and ChemSpider Search; and S/N Threshold: 3. Finally, according to the software search report, combined with fragment information, the structures of 165 components were speculated, of which 20 were further confirmed with reference substances. The components in JZDHW included anthraquinones, anthrones, tannins, phenbutyl ketones, and corresponding glycosides. Among them, free and bound anthraquinones were the main components, including emodin, rhein, aloe-emodin, and their glycosides.

### Analysis of Metabolites in Human Plasma

**FIGURE 2:**
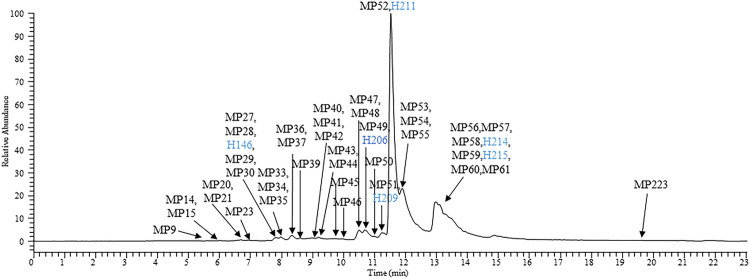
High-resolution extracted ion chromatogram of the main exposed components of human plasma samples processed using the methanol protein precipitation method. Blue: prototype components of rhubarb.

A variety of targeted and non-targeted intelligent MS data post-processing technologies (Wu et al., 2016; Zhu et al., 2020; Chen T et al., 2021; Chen X et al., 2021), such as high resolution extraction ion chromatography (HREIC), precise-and-thorough background-subtraction, mass defect filter, and data-independent acquisition-product-ion filter, have been used in recent years to establish a research strategy for the comprehensive identification of TCM-related compounds *in vivo*. The present study directly searched the prototype and metabolite components of JZDHW from the perspective of the human body based on the technical strategy developed in the early stage. For the first time, 72 JZDHW-related compounds were identified in human plasma samples, including 11 prototype components, such as rhein, emodin, and gallic acid, and 61 related metabolites, including primarily glucuronidated and sulphated metabolites (Supplementary Table S2) (Song et al., 2010; Zhu et al., 2015; Huang et al., 2019; Xu et al., 2019). Figure 2 shows an extracted ion chromatogram of human plasma samples after completion of the methanol precipitation method. In addition, Supplementary Figure S1 shows an extracted ion chromatogram of human plasma samples pre-treated with solid phase extraction.

### Semi-Quantitative Determination of Exposure of JZDHW-Related Compounds in Human Plasma as Determined Using the AUC Pooled Method

**FIGURE 3:**
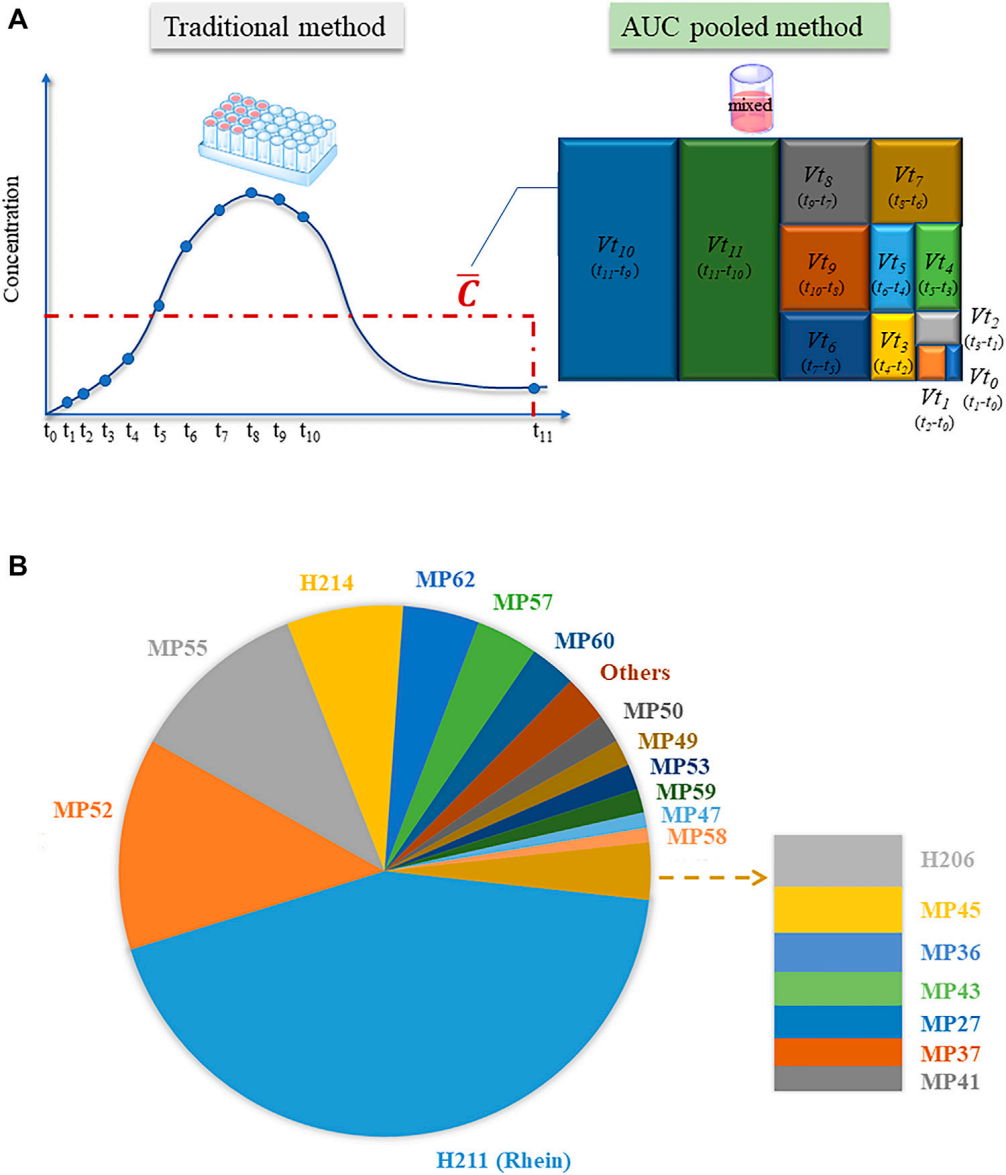
Research on the area under the time-concentration curve (AUC) of relevant components *in vivo*. **(A)** Operational differences between the traditional and AUC pooled methods; **(B)** Top 20 rhubarb-related components *in vivo*.

Traditionally, the exposure of compounds *in vivo* is obtained by measuring the drug concentration at multiple time points to draw a drug-time curve, and then obtaining exposure parameters such as AUC. For TCM, this method has some improvements: the components of TCM are complex, and it is difficult to quantify multiple components; more importantly, many components cannot be obtained as reference materials, thus exposure data cannot be quantitatively obtained. In this study, we applied the AUC pooled method to study the exposure of TCM for the first time. Using the formula $v_{1}∶v_{2}∶\cdots ∶v_{i}=\left(t_{1}-t_{0}\right)∶\left(t_{2}-t_{0}\right)∶\left(t_{3}-t_{1}\right)∶\cdots ∶\left(t_{i}-t_{i-1}\right)$ , we calculated the volume ratio of plasma at different time points, and then mixed the plasma samples. The measured concentration (indicated by the peak area without reference substance) multiplied by the last collection time value directly represents the exposure of prototype and metabolite components *in vivo* (as shown in Figure 3A). Taking rhein as an example, a combination of the traditional and AUC pooled methods was used to determine the AUC values of the human and rat plasma samples. As shown in Table 1, the difference between the two methods was 12.78% in rat samples and 0.58% in human samples, both of which are within 15%, indicating that the AUC pooled method can effectively replace the traditional method to obtain more accurate AUC values.

**TABLE 1:** Determination of the area under the time-concentration curve (AUC) using the AUC pooled and traditional methods.

	Rat-rhein	Human-rhein
No pooled-AUC(mg/L*h)	72517.5 ± 42.2	57651 ± 47.8
AUC-pooled (mg/L*h)	81786.81 ± 24.7	57984.4 ± 19.3
Difference	12.78%	0.58%

In this study, the human plasma collected at each time point was mixed and measured according to the AUC pooled method. The exposure difference in human plasma was visually shown using peak area data from HREIC. Among the 20 compounds with high exposure, H211 (rhein), M52 (oxidized aloe emodin), MP54 (sulphated emodin isomer), and H214 (laccaic acid D) were the main substances *in vivo*, followed by the sulphated metabolites of anthraquinone compounds such as emodin, chrysophanol, aloe-emodin, and rhein (Figure 3B). It is worth mentioning that the exposure of the top ten components accounted for 90% of the exposure of all compounds *in vivo*. Among the top ten exposed components, only H211 (rhein) could be obtained as a reference substance, and therefore could be considered as a potential Q-marker of JZDHW.

**FIGURE 4:**
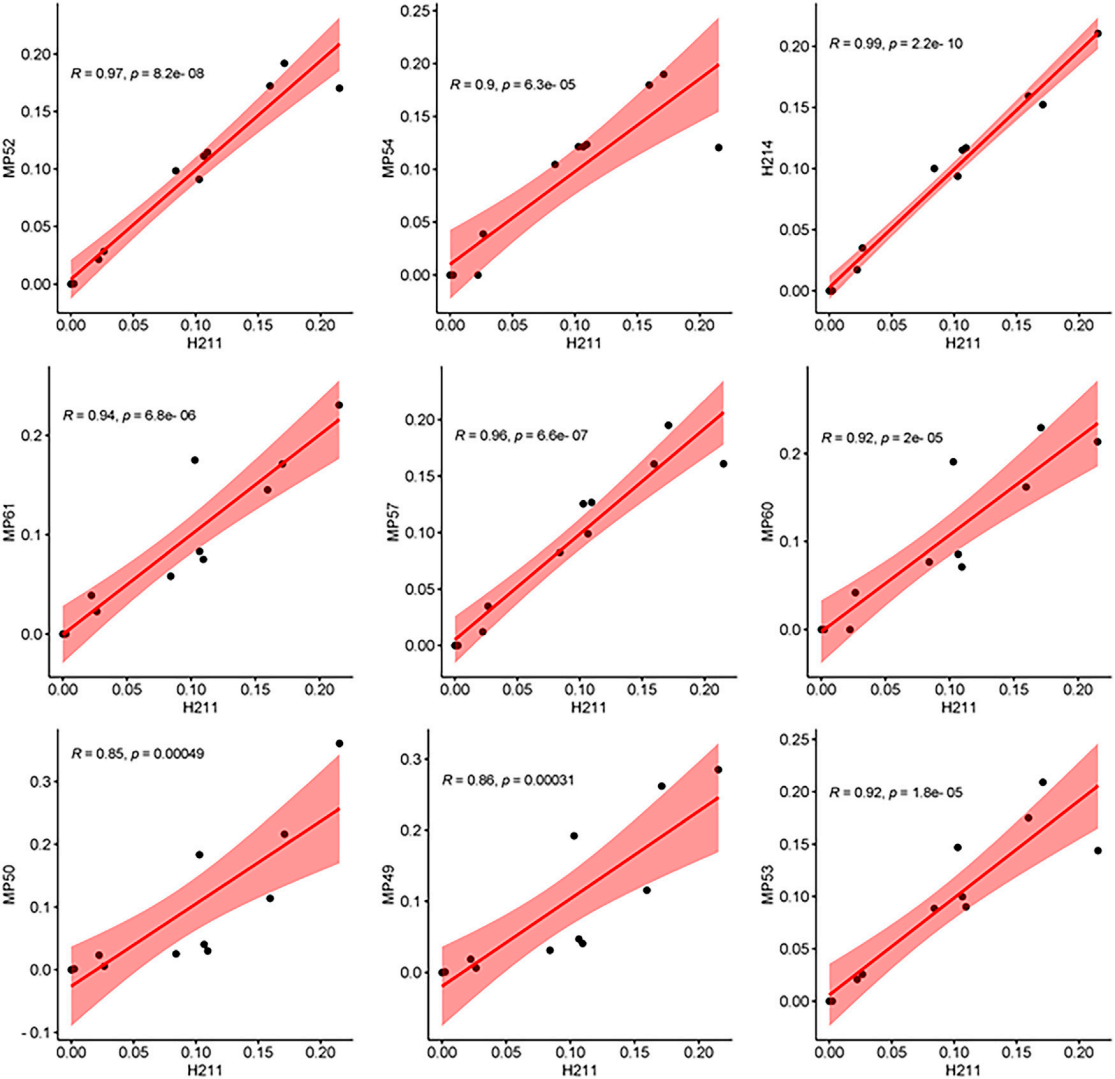
Pearson correlation analysis between the main exposed compounds and rhein.

To further determine whether rhein can reflect or predict the overall metabolic trend of JZDHW anthraquinone and its metabolites in the human body, we compared the percentage concentrations of the nine other top components at different collection time points with those of rhein using Pearson correlation coefficient analysis (Figure 4). We found that all nine components had a strong correlation with rhein (0.8 < R < 1, *p* < 0.001). Therefore, rhein could be selected as a Q-marker to reflect the pharmacokinetic trend of anthraquinone components of JZDHW in humans.

### Selection of Pharmacodynamic Model Animals

**FIGURE 5:**
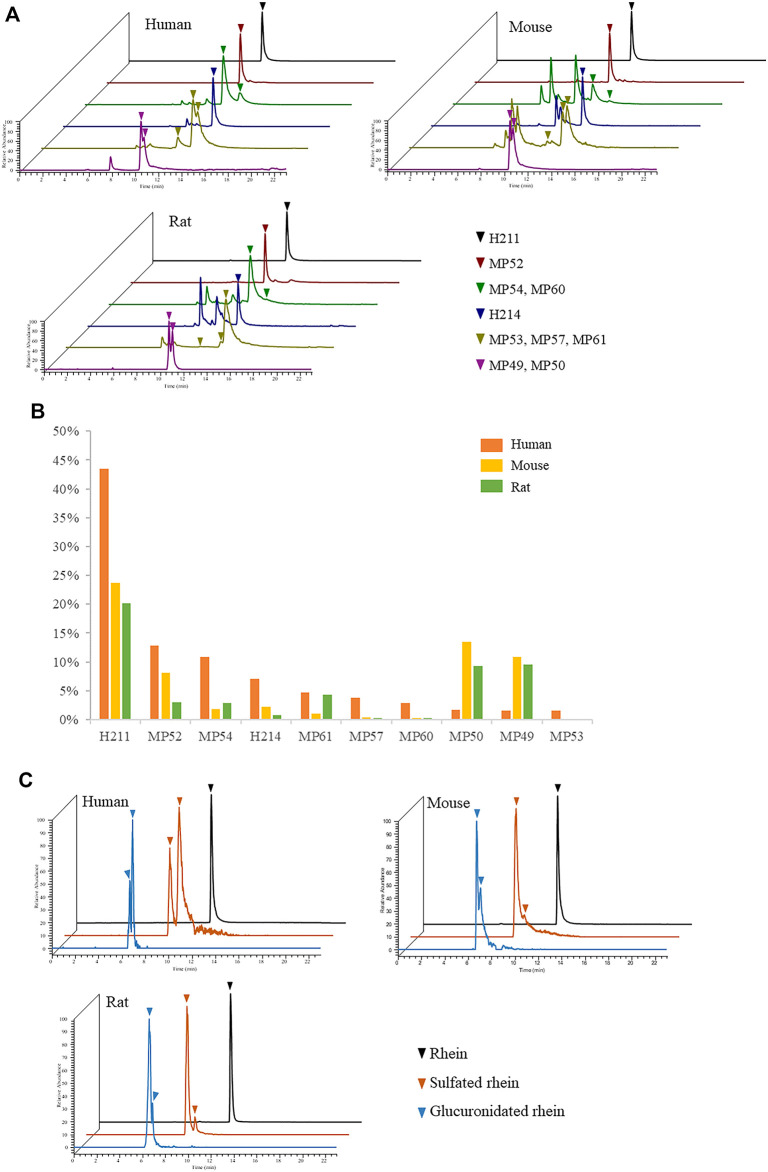
Metabolism of rhubarb in humans, rats, and mice. **(A)** Exposure of the top 10 compounds in human exposure in different species; **(B)** Proportion of the top 10 compounds in human exposure in different species; **(C)** Metabolism of rhein in different species.

Animals such as rats and mice are commonly used in the study of TCM pharmacodynamics. However, metabolism varies in different species. Only on the basis of verifying the metabolism consistency of animals and humans, the obtained pharmacodynamic data had more reference significance for clinical research. We analysed the plasma samples of SD rats and C57BL/6 mice after oral administration of JZDHW solution and searched for 192 JZDHW-related compounds (Supplementary Table S2). The top 10 compounds in human exposure were also well exposed in rats and mice (Figure 5A), accounting for 51 and 62% of all exposed compounds in their bodies, respectively (Figure 5B). Mice were slightly better than rats. It is worth mentioning that the exposure of rhein was the highest in all three species. Moreover, the main metabolites of rhein-glucuronidated and sulphated metabolites could be found in rodents (Figure 5C). In Figure 5C, rats, mice, and human had different metabolic binding sites. No matter that it was sulfation or glucuronidation, both humans and rodents had their own preferred metabolic sites, which might be caused by difference types of metabolic enzymes. In addition, comparing the total peak areas of rhein sulfated metabolites with those of glucuronidated metabolites in three species, it was found that sulfation was more preferred in humans while glucuronidation was more preferred in mice and rats. This finding may be caused by differences in the activity of metabolic enzymes. In general, in terms of metabolic pathways and binding sites, there were no significant differences between rats and mice. However, the main exposed compounds of JZDHW in humans can be well exposed in rats and mice. In short, rodents can also be used as a suitable model animal to study the efficacy of rhubarb.

### Pharmacodynamic Verification

**FIGURE 6:**
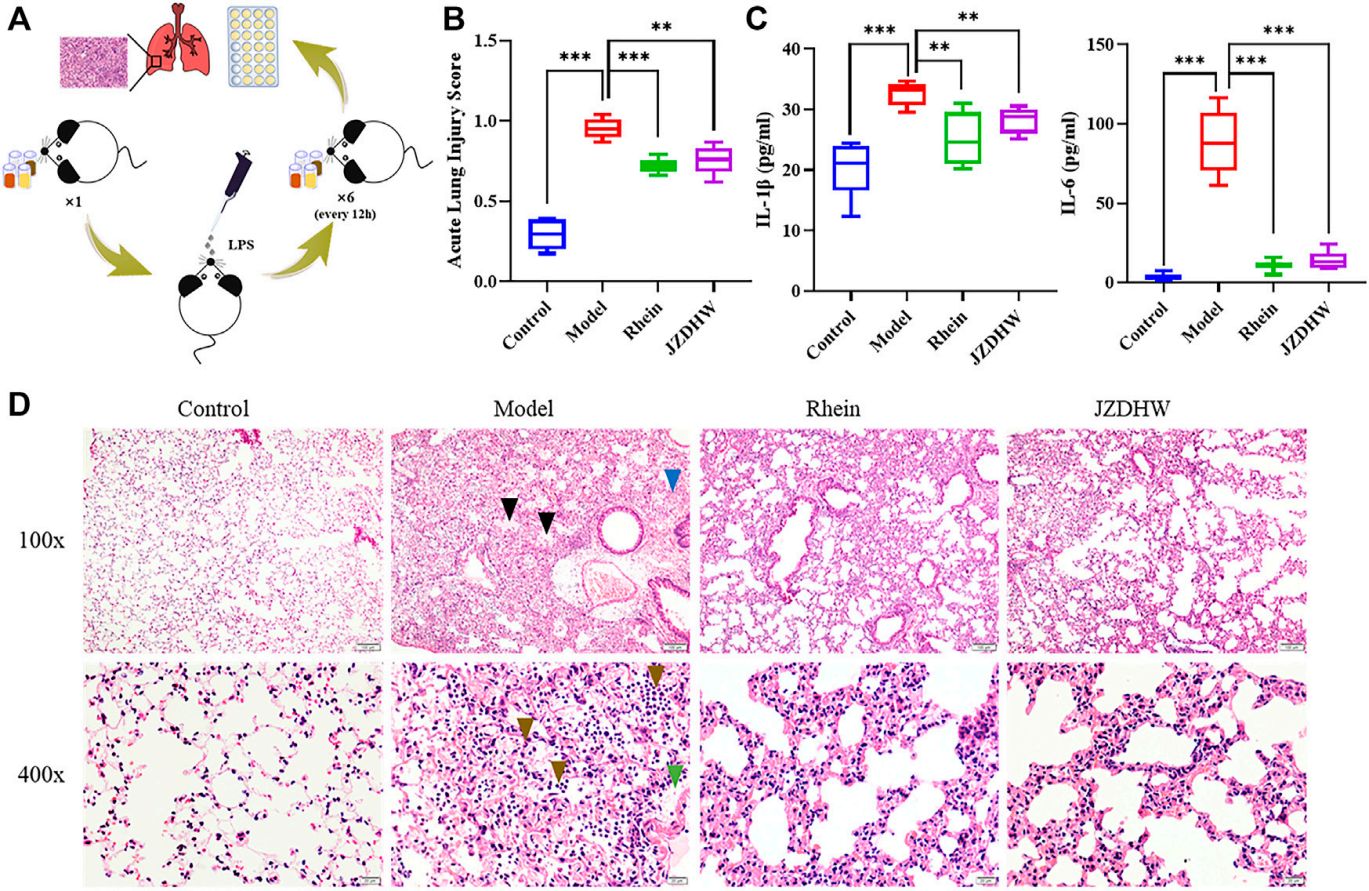
Therapeutic effect of rhubarb and its monomers on pneumonia. **(A)** Schematic diagram of the experiment; **(B)** Acute lung injury score; **(C)** Pro-inflammatory factor ELISA test; **(D)** Tissue section (100 × magnification and ×400). 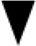
indicates oedema, 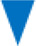
indicates bleeding, 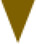
indicates inflammatory cells, and 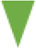
indicates fibrin oozing out of the alveoli. The results are given as mean ± standard error of the mean (*n* = 6).

Pneumonia is associated with inflammatory stimulation from microorganisms, such as endotoxin, which causes severe pneumonia. As a potent endotoxin, lipopolysaccharide is used by many scholars to induce the establishment of an animal model of pneumonia to evaluate the efficacy of the drug in recent years. This is a relatively simple, efficient, safe, low-cost, and mature method. Based on LPS-induced pulmonary inflammation in C57BL/6 mice, four groups were established to compare the effects of pneumonia treatment: the control, model, rhein, and JZDHW groups. In clinical treatment, the human dosage of JZDHW is 6 g/d. By considering the conversion based on body surface area of human (70 kg) and mice (0.02 kg), the dose for mice was about 0.0156 g/d. In order to ensure the pharmaceutical efficacy, a double dosage was applied. Therefore, the dose of JZDHW was determined as 1.6 g/kg. The dose of rhein was consistent with that of total anthraquinone in JZDHW, which was calculated according to the 2020 edition of Pharmacopoeia of the People’s Republic of China. In details, the dose of rhein was 85 mg/kg. After the animal experiments (Figure 6A), we analysed the pathological changes in the lung tissue and changes in the serum pro-inflammatory factors. Inflammatory cell infiltration, alveolar haemorrhage, interstitial oedema, thickening of the alveolar septum, and fibrin exudation in the alveoli are the main features of LPS-induced pneumonia. According to the histopathological characteristics of the acute lung injury scoring system (Li et al., 2019) (Figure 6B), the scores of the therapy groups were significantly reduced compared with those in the model group. Furthermore, the oedema of the therapy groups was significantly relieved, and the infiltration of inflammatory cells was significantly reduced (*p* < 0.01) (Figure 6D). In addition, the concentrations of serum pro-inflammatory factors IL-1β and IL-6 in the therapy groups were significantly lower than those in the model group (Figure 6C). Combined with the current epidemic COVID-19, the clinical studies have shown that cytokine storms were prone to occur in infected patients, leading to inflammation, infiltration of macrophages, neutrophils, and multiple organ damage. More importantly, IL-6 was one of the key inflammatory factors. Therefore, cytokine storm can be induced by LPS stimulation to simulate that of COVID-19. In summary, JZDHW and its monomer (rhein) can effectively alleviate the symptoms of acute pneumonia and can be used as candidate drugs for COVID-19.

## Discussion

**FIGURE 7:**
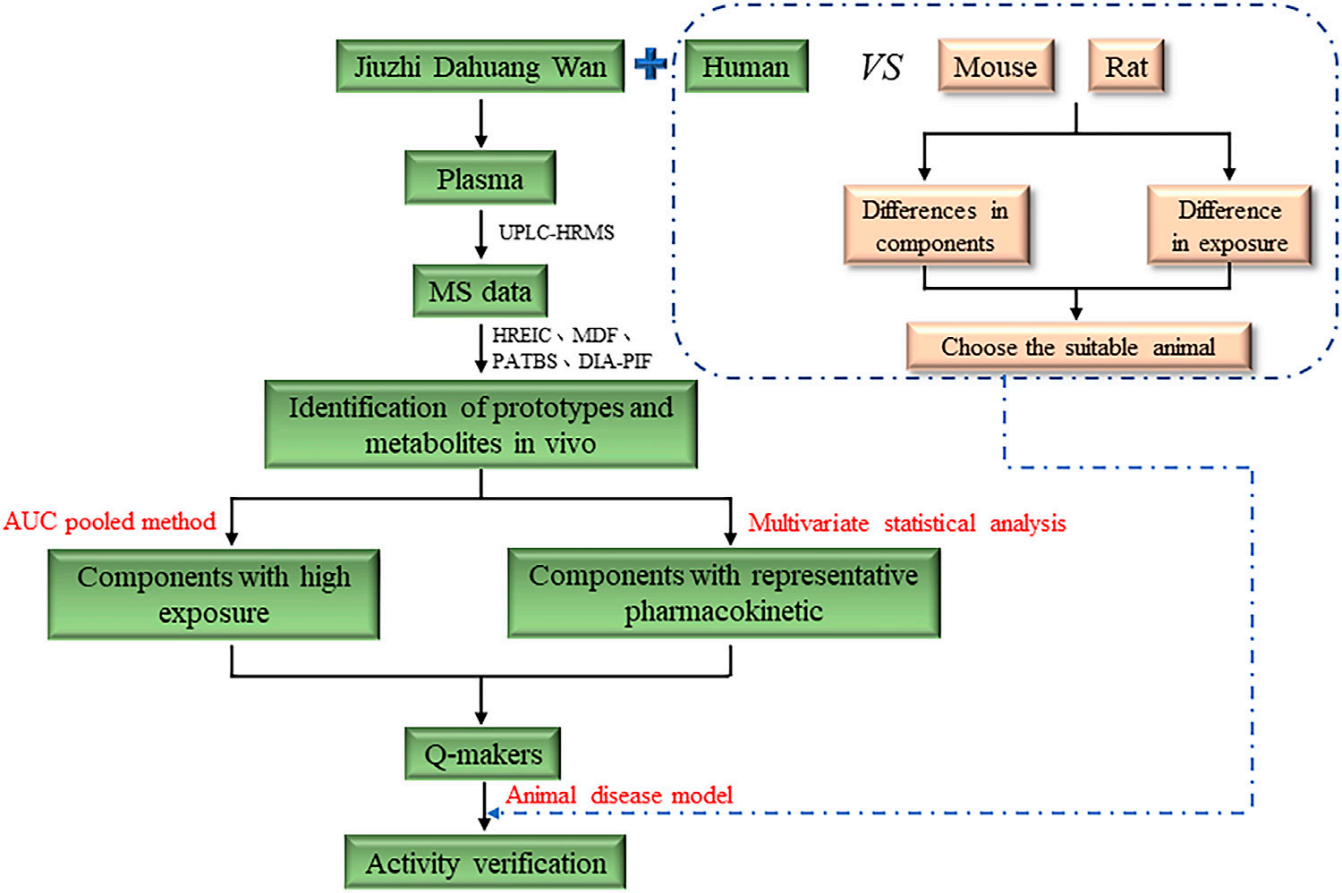
Workflow of the research strategy.

From the perspective of the human body, this study used the laboratory’s self-built data post-processing system to search and identify prototype and metabolite compounds of JZDHW by UPLC-HRMS. We used the simple and practical AUC pooled method to quickly obtain the exposure of each component and screened out the main exposed substances. We then performed correlation analysis on the pharmacokinetic curve to find a representative Q-marker, rhein (Figure 7). The results showed that the AUC pooled method can effectively aid in the quick determination of potential Q-markers of TCM.

**FIGURE 8:**
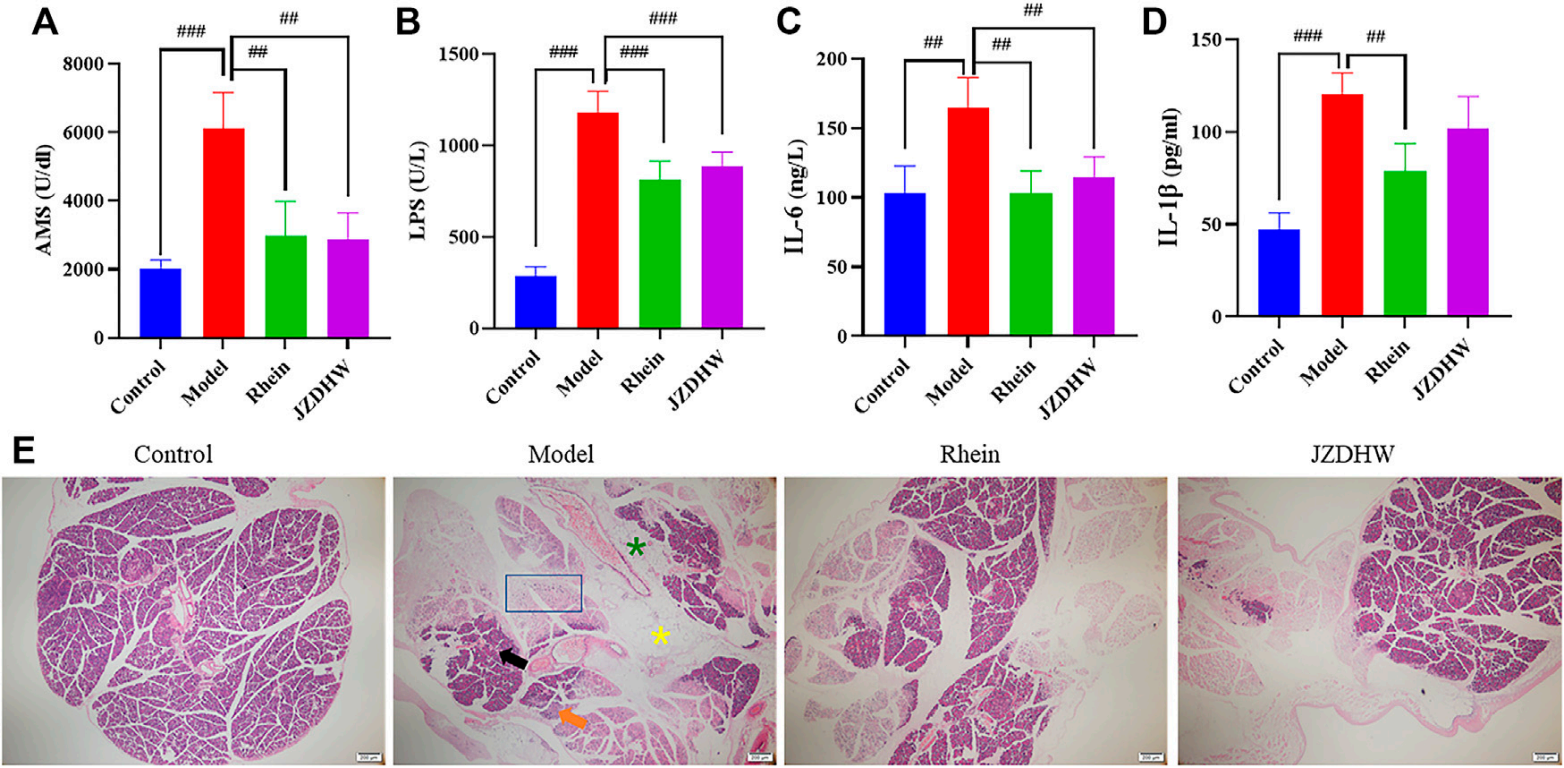
Therapeutic effect of rhubarb and its monomers on pancreatitis. **(A)** Serum amylase; **(B)** Lipase; **(C)** IL-6; **(D)** IL-1β; **(E)** Tissue section (40 × magnification), 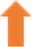
indicates haemorrhage, 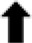
indicates oedema, 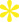
indicates fatty acid, 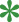
indicates inflammatory cells, and 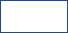
indicates necrosis. The results are given as mean ± standard error of the mean (*n* = 6). Significant differences between groups are represented by # for *p* < 0.05, ## for *p* < 0.01 and ### for *p* < 0.001.

Before establishing an animal model to evaluate pharmacodynamics, it should be verified that the main prototype and metabolite compounds in the chosen animal are consistent with those in humans. The exposure difference between humans and animals is often ignored by studies on the efficacy of TCM. This study considered the possibility of the existence of this difference, and experimentally proved that the Q-marker rhein is retained in the plasma of SD rats and C57BL/6 mice, and generated glucuronidated and sulphated metabolites, which were similar to those in humans. This guaranteed the reliability of our animal model selection. In addition to the acute pneumonia model, we also conducted an efficacy verification in an acute pancreatitis model (the dosages for both models were determined by the same principle). The results showed that rhein could significantly reduce the concentration of serum amylase and lipase, which are diagnostic indicators of acute pancreatitis, and reduce pathological symptoms such as bleeding, oedema, necrosis, and inflammatory cell infiltration. The concentration of pro-inflammatory factors IL-6 and IL-1β were also reduced, indicating a positive anti-inflammatory effect (Figure 8). In this study, based on human exposure and the AUC pooled method, the anthraquinone monomer rhein with high exposure *in vivo* and a significant anti-inflammatory effect was recorded. More importantly, rhein can represent the overall metabolic changes of JZDHW anthraquinone in humans and rodents.

Therefore, based on the Chinese Pharmacopoeia and our research data, we believed that, besides to the monitoring of the total content of total anthraquinones and free anthraquinones, it was also necessary to control the content of rhein individually, because of its high content in rhubarb, good pharmacokinetic properties, and good pharmacological effects *in vivo*. However, there are many anthraquinone compounds in rhubarb, which may be mutual conversion *in vivo*, leading interference to formulation of the quality standard range of Q-marker. Our previous studies had shown that chrysophanol and aloe-emodin could be converted into rhein in rats. However, the amount of rhein converted from other anthraquinone compounds *in vivo* was little, because chrysophanol and aloe-emodin also underwent glucuronidation, sulfation and other reactions. And the application of this phenomenon to humans was uncertain. In addition, from the perspective of drug interactions, other anthraquinone compounds, which had an effect on the absorption and metabolism of rhein, still needed to be further elucidated. Therefore, it was necessary to combine the influence of other anthraquinones, when determining the quality control range of rhein.

## Conclusion

Through the pharmacokinetics exploration of rhubarb in humans for the first time based on the AUC pooled semi-quantitative method and the pharmacodynamic verification of different animal models, rhein can be experimentally proved to be Q-marker of rhubarb, whose content needs to be strictly controlled. Furthermore, the research strategy established in this study might also be beneficial in the identification of Q-markers in other TCM, and provide a reference for the improvement of quality standards, so as to carry out stricter quality control of TCM.

## Data Availability

The original contributions presented in the study are included in the article/Supplementary Material, further inquiries can be directed to the corresponding author.

## Abbreviations

AUC, Area Under the Time-concentration Curve; CMC, Carboxymethyl Cellulose; HREIC, High Resolution Extraction Ion Chromatography; IL, Interleukin; JZDHW, Jiuzhi Dahuang Wan; LPS, Lipopolysaccharide; Q-marker, Quality-markers; SD, Sprague Dawley; TCM, Traditional Chinese Medicine; tm, Time; TNFα, Tumour Necrosis Factor Alpha; UPLC-HRMS, Ultra performance Liquid Chromatography-High Resolution Mass Spectrometry.
