# Supplementary material for: Exploration of Q-Marker of Rhubarb Based on Intelligent Data Processing Techniques and the AUC Pooled Method
Source: Front Pharmacol. 2022 Mar 21;13:865066. doi: 10.3389/fphar.2022.865066 (PMC8979112; doi:10.3389/fphar.2022.865066)
Supplement: Supplementary file 1 [file DataSheet1.docx]

Supplementary Material

# Supplementary Methods

## Development and verification of the AUC pooled method

Standard curve preparation: We prepared 720 μg/mL rhein mother liquor with methanol, and then diluted the preparation to a series of standard solutions with concentrations of 240, 72, 24, 7.2, 2.4, and 0.72 μg/mL. Then, we added 10 μL of each concentration of standard working solution to 470 μL blank plasma, respectively. Based on a single matrix of 240 μL plasma, plasma standard curve samples with different concentrations (30 μg/mL, 10 μg/mL, 3 μg/mL, 1 μg/mL, 300 ng/mL, 100 ng/mL, and 30 ng/mL) were obtained. The protein was then precipitated with three times the volume of methanol. After centrifugation, the supernatant was blown dry with nitrogen. The residues were reconstituted with 100 μL of 70% methanol for testing.

Traditional method: A total of 240 μL of rat plasma was collected at 12 time points (0, 0.25, 0.5, 1, 2, 3, 4, 6, 8, 10, 12, and 24 h) to obtain a total of 12 samples. The samples were then added to 240 μL of blank human plasma. The protein was precipitated with three times the volume of methanol. After centrifugation, the supernatant was blown dry with nitrogen. The residues were reconstituted with 100 μL of 70% methanol for testing. Each set of samples was evaluated in triplicate. The protocol for pre-treatment of human plasma samples was the same as that described above, except that 240 μL of blank rat plasma was added instead of 240 µL of blank human plasma.

AUC pooled method: Rat plasma samples (1.25, 2.5, 3.75, 7.5, 10, 10, 15, 20, 20, 20, 70, and 60 μL) were collected at 12 time points and mixed uniformly to obtain 240 μL mixed plasma. The mixed sample was then added to 240 μL of blank human plasma. The protein was precipitated with three times the volume of methanol. After centrifugation, the supernatant was blown dry with nitrogen. The residues were reconstituted with 100 μL of 70% methanol for testing. Each set of samples was in triplicate. The protocol for pre-treatment of human plasma samples was the same as above, except that 240 μL of blank rat plasma was added in place of the 240 µL of blank human plasma.

UPLC-HRMS analysis: The samples were analysed using a Thermo Fisher Q Exactive Orbitrap liquid chromatography with tandem mass spectrometry system equipped with electrospray ionization (Thermo Fisher Scientific) in negative ion mode, which was controlled by Thermo Xcalibur 3.0.63 (Thermo Fisher Scientific). An ACQUITY ultra performance liquid chromatography CSH C18 column (50 mm × 2.1 mm, 1.7 μm; Waters Corporation) was used to separate the sample at a temperature of 35 °C. Mobile phase A was H_2_O with 0.1% formic acid, and mobile phase B was 100% acetonitrile with a flow rate of 0.3 mL/min. The injection volume was set at 3 μL. The mobile phase gradient was set as follows: 0–5 min, 70%–5% (A); 5–7 min, 5%–5% (A); 7–7.5 min, 5%–70% (A); and 7.5–11 min, 70%–70% (A). The MS parameters were set as follows: full MS resolution 70000, scanning range *m/z* 100–1000, spray voltage 3.5 kV, capillary temperature 320 °C, sheath gas (N2) flow rate 35 arb, auxiliary gas (N2) flow rate 10 arb, and sweep gas (N2) flow rate 5 arb.

## Pre-treatment by solid phase extraction

Human/rat plasma samples: Samples (50, 100, 150, 300, 400, 400, 600, 800, 800, 800, 2800, and 2400 μL) from the mixed plasma collected at 0, 0.25, 0.5, 1, 2, 3, 4, 6, 8, 10, 12, and 24 h after oral administration of rhubarb solution, respectively, were mixed to obtain 9.6 mL plasma. The samples were diluted with 4% phosphoric acid in equal volume before passing through 6cc HLB cartridges. The cartridges were activated with 4 mL methanol and 4 mL pure water. The sample was then loaded, rinsed with 4 mL water, eluted with 4 mL methanol, and the elution was collected. Finally, the sample was dried using nitrogen. The residues were reconstituted with 100 μL of 70% methanol.

Mice plasma samples: Samples (15, 30, 45, 90, 120, 120, 180, 240, 240, 240, 840, and 720 μL) from the mixed plasma collected at 0, 0.25, 0.5, 1, 2, 3, 4, 6, 8, 10, 12, and 24 h after oral administration of rhubarb solution, respectively, were mixed to obtain 2880 μL plasma. The samples were diluted with 4% phosphoric acid in equal volume before passing through 3cc HLB cartridges. The cartridges were activated with 2 mL methanol and 2 mL pure water. The samples were then loaded, rinsed with 2 mL water, eluted with 2 mL methanol, and the elution was collected. The sample was then dried using nitrogen. The residues were reconstituted with 100 μL of 70% methanol.

## Pharmacodynamic verification of the pancreatitis model

A total of 24 female Sprague Dawley rats, 180–220 g, were purchased from Shanghai SLAC Laboratory Animal Co., Ltd. (Shanghai, China), and randomly divided into five groups: the control group (A), model group (B), rhein group (C), and JZDHW group (D). Mice were adaptively fed for one week prior to starting the experiment. Before modelling, groups A and B were administered 0.4% CMC, group C was administered 60 mg/kg rhein, and group D was administered 1.1 g/kg JZDHW three times (36, 12, and 0 h before modelling), with a volume of 20 mL/kg. All treatments were administered by oral gavage. Immediately after pre-treatment, an acute pancreatitis model was established by retrograde pancreaticobiliary injection of 4% sodium taurocholate. Rats were anesthetized by intraperitoneal injection of chloral hydrate. After 6 h, the rats were sacrificed, and serum and pancreatic tissue were collected. ELISA kits were used to detect the changes in serum inflammatory factors (IL-6 and IL-1β), serum amylase, and lipase. A Rat IL-1β ELISA kit was purchased from eBioscience (San Diego, CA, USA), and rat IL-6, serum amylase, and lipase ELISA kits were purchased from Nanjing Jiancheng Institute of Biological Engineering (Nanjing, China). GraphPad Prism 8.0.2 software (GraphPad Software, San Diego, CA, USA) was used for statistical analysis. Significance was analysed using a two-tailed Student's t-test and one-way analysis of variance. Significant differences between groups are represented by # for p <0.05, ## for p <0.01 and ### for p <0.001. Haematoxylin and eosin staining was used to analyse the pathological conditions of the pancreatic tissue.

# Supplementary Figures and Tables

## Supplementary Figures


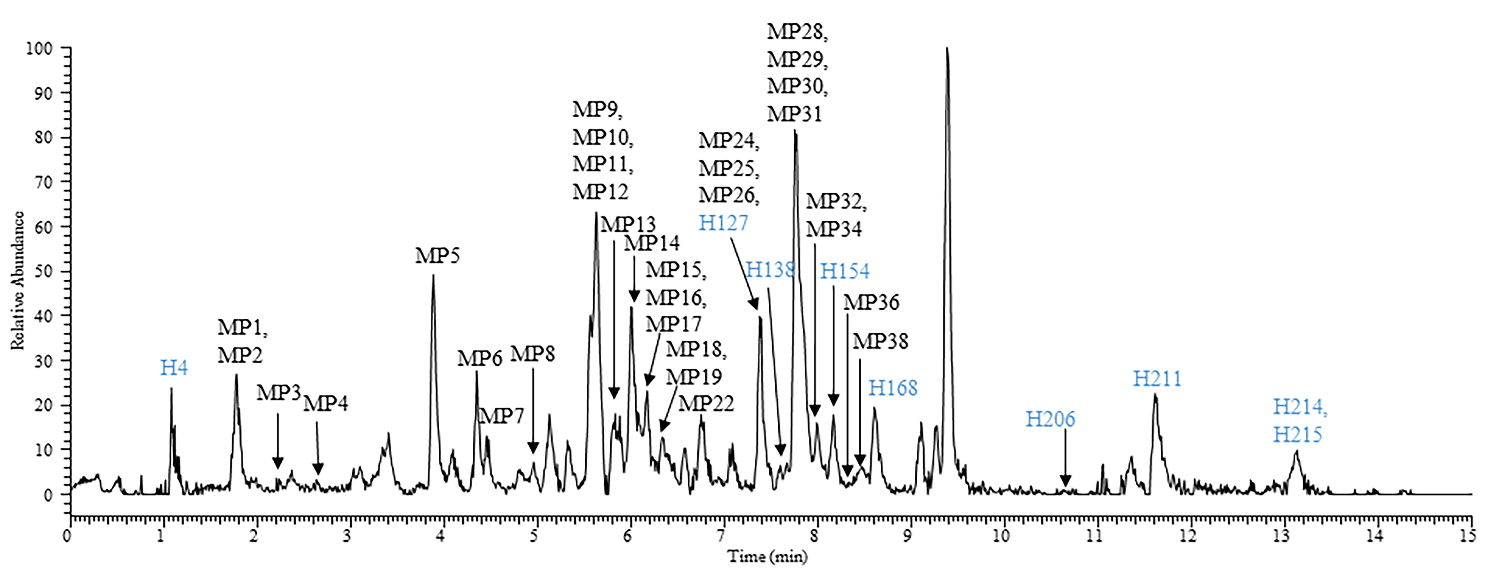


**Supplementary Figure 1.** High-resolution extracted ion chromatogram of the main exposed components of human plasma samples processed by solid phase extraction. Blue: prototype components of rhubarb

## Supplementary Tables

**Supplementary Table 1.** Ingredients of Rhubarb

| No. | RT | HRMS- | Formula | Error(ppm) | MS2 | Compounds |
| --- | --- | --- | --- | --- | --- | --- |
| H1 | 0.58 | 331.06732 | C13H15O10 | 0.756 | 331.06821(11),211.02481(28),169.01370(100),125.02350(18) | Glucosyl-gallic acid |
| H2 | 0.83 | 191.01894 | C6H7O7 | -4.114 | 191.01938(7),111.00768(100),87.00751(48) | Citric acid |
| H3 | 0.90 | 331.06726 | C13H15O10 | 0.574 | 331.06842(8),169.01373(100),125.02348(19) | Glucosyl-gallic acid |
| H4* | 1.08 | 169.01320 | C7H5O5 | -6.192 | 169.01370(36),125.02340(100) | Gallic acid |
| H5 | 1.23 | 287.07727 | C12H15O8 | 0.102 | 189.00374(2),125.02349(100),110.02371(10) |  |
| H6 | 1.33 | 345.08301 | C14H17O10 | 1.387 |  | Methyl-glucosyl-gallic acid |
| H7 | 1.50 | 248.97105 | C7H5O8S | -0.044 | 248.97166(30),230.96077(100),125.02342(22) | Sulfated Gallic Acid |
| H8 | 1.55 | 517.08398 | C20H21O16 | 0.914 | 169.01326(76),125.02354(4) |  |
| H9 | 1.67 | 331.06735 | C13H15O10 | 0.846 | 169.0137(100),125.02345(25) | Glucosyl-gallic acid |
| H10 | 2.23 | 451.12509 | C21H23O11 | 1.120 | 289.07275(100),245.08247(49),203.07143(16),137.02353(24),125.02355(17) | Glucosyl catechin |
| H11 | 2.62 | 183.02890 | C8H7O5 | -5.445 | 183.03194(100),168.00546(97),139.0388(14),124.01533(61) | Methylgallic acid |
| H12 | 2.64 | 219.05063 | C8H11O7 | -1.807 | 157.04956(9),111.00742(100) | Dimethy citric acid |
| H14 | 2.76 | 183.02873 | C8H7O5 | -6.374 | 183.03212(37),168.00548(100),124.01534(47) | Methylgallic acid |
| H13 | 2.76 | 345.08493 | C14H17O10 | 3.307 | 183.02911(100) | Methyl-glucosyl-gallic acid |
| H15 | 3.01 | 451.12518 | C21H23O11 | 1.320 | 289.07275(100),245.08247(49),137.02353(24),125.02355(17) | Glucosyl catechin |
| H16 | 3.05 | 325.09320 | C15H17O8 | 0.951 | 187.03932(61),161.05983(41),145.02844(100),119.0489(71) | Glucosyl-coumaric isomers |
| H17 | 3.17 | 373.07797 | C15H17O11 | 0.899 | 373.07895(3),169.01369(100),125.02348(24) | Acetyl glucosyl gallic acid |
| H18 | 3.23 | 183.02907 | C8H7O5 | -4.516 | 183.02907(100),168.00542(36),124.01530(27) | Methylgallic acid |
| H19 | 3.30 | 229.09795 | C13H13O2N2 | -1.314 | 185.10708(54),142.06546(34) | 1-Methyl-1, 2, 3, 4-tetrahydro-β-carboline-3-carboxylic acid or isomers |
| H20 | 3.33 | 451.12509 | C21H23O11 | 1.120 | 289.07275(100),245.08244(50),203.07121(15),125.02339(15) | Glucosyl catechin |
| H21 | 3.34 | 325.09320 | C15H17O8 | 0.951 | 187.03922(63),161.05968(25),145.02829(100),119.0489(31) | Glucosyl-coumaric acid |
| H22 | 3.50 | 248.97115 | C7H5O8S | 0.357 | 169.01370(100),125.02350(17) | Sulfated Gallic Acid |
| H23 | 3.54 | 483.07864 | C20H19O14 | 1.266 | 313.05685(28),271.04620(93),211.02426(42),169.01329(100),125.02329(15) | Digalloyl-D-glucose |
| H24* | 3.55 | 289.07170 | C15H13O6 | 1.206 | 289.07280(48),245.08246(82),203.07120(82),125.02340(86),109.02840(100) | Catechin |
| H25 | 3.65 | 325.09320 | C15H17O8 | 0.951 | 187.03912(7),163.03896(6),145.02834(100),119.04890(7) | Glucosyl-coumaric acid |
| H26 | 3.71 | 229.09799 | C13H13O2N2 | -1.139 | 185.10786(99),183.09212(19),116.04929(50) | 1-Methyl-1, 2, 3, 4-tetrahydro-β-carboline-3-carboxylic acid or isomers |
| H27 | 3.71 | 645.13171 | C26H29O19 | 1.330 | 475.10965(7),313.05652(56),169.01326(100),125.02333(11) |  |
| H28 | 3.73 | 517.15662 | C22H29O14 | 0.660 | 193.05048(11),175.03963(100),160.01596(23) |  |
| H29 | 3.77 | 165.05478 | C9H9O3 | -5.680 | 165.05508(100),149.02365(11),123.04416(25),121.06487(71) |  |
| H30 | 4.09 | 483.07840 | C20H19O14 | 0.769 | 313.05676(18),169.01328(100),125.02314(19) | Digalloyl-D-glucose |
| H31 | 4.21 | 397.11420 | C18H21O10 | 0.453 | 233.08266(5),189.05548(100) |  |
| H32 | 4.21 | 451.08865 | C20H19O12 | 0.999 | 313.05765(60),169.01373(100),137.02356(42) | 2-O-(4-Hydroxybenzoyl)-6-O-(3,4,5-trihydroxybenzoyl)-D-glucopyranose or isomers |
| H33 | 4.22 | 325.09308 | C15H17O8 | 1.286 | 163.03903(100),161.05956(41),145.02834(75),119.04895(52) | Glucosyl-coumaric acid |
| H34 | 4.26 | 373.07779 | C15H17O11 | 0.417 | 169.01375(100),125.02346(32) | Acetyl glucosyl gallic acid |
| H35 | 4.29 | 493.13589 | C23H25O12 | 1.502 | 289.07233(100),245.08092(49),169.01341(35),125.02312(26) | Acetyl glucosyl-catechin |
| H36 | 4.48 | 517.15692 | C22H29O14 | 1.240 | 193.05040(84),175.03961(100),134.03647(37) |  |
| H37 | 4.55 | 593.15204 | C27H29O15 | 1.428 | 311.05725(3),269.04517(88),268.03796(100) | Diglucosyl-emodin |
| H38 | 4.56 | 479.12000 | C22H23O12 | 1.045 | 255.06624(100),167.03383(5),149.02330(21) |  |
| H39 | 4.59 | 547.11017 | C25H23O14 | 1.538 | 313.0575(59),189.0555(60),169.01370(100),151.00307(12),125.02359(16) |  |
| H40 | 4.67 | 248.97112 | C7H5O8S | 0.237 | 248.97176(3),169.01370(100),125.02346(16) | Sulfated Gallic Acid |
| H41 | 4.67 | 559.16797 | C24H31O15 | 2.015 | 175.03961(100),160.01595(29) |  |
| H42 | 4.70 | 593.15222 | C27H29O15 | 1.731 | 293.04459(3),269.04581(100),268.03836(7),240.04242(4) | Diglucosyl-aloe emodin |
| H43 | 4.74 | 493.13583 | C23H25O12 | 1.380 | 289.07193(100),245.08192(50),205.04935(17),203.07054(17),125.02300(22) | Acetyl glucosyl-catechin |
| H44 | 4.81 | 197.04492 | C9H9O5 | -3.180 | 197.04567(100),169.01364(44),125.02360(17) | Dimethylgallic acid |
| H45 | 4.84 | 451.08926 | C20H19O12 | 2.352 | 451.13101(9),313.05756(51),271.04669(13),169.01370(100),125.02349(15) | 2-O-(4-Hydroxybenzoyl)-6-O-(3,4,5-trihydroxybenzoyl)-D-glucopyranose or isomers |
| H46 | 4.92 | 165.05472 | C9H9O3 | -6.043 | 165.05507(36),147.04440(100) | Paeonol |
| H47 | 5.00 | 547.14655 | C26H27O13 | 1.528 | 313.05756(95),233.08246(31),189.05537(18),169.01369(100) | 2-(2'-Hydroxypropyl)-5-methyl-7-hydroxychromone-glucosyl-gallic acid |
| H48 | 5.02 | 581.22479 | C28H37O13 | 1.421 | 419.17261(71),404.14850(74),373.13037(92) | Lyoniresinol-3a-O-β-glucoside or isomers |
| H49 | 5.11 | 371.17139 | C18H27O8 | 0.671 | 371.17139(67),209.11783(100) | (2S)-4-[(4-Methoxybenzyl)oxy]-2-butanyl β-D-glucopyranoside or isomers |
| H50* | 5.15 | 163.03905 | C9H7O3 | -6.240 | 163.03946(11),119.04922(100) | p-Coumaric acid |
| H51 | 5.17 | 189.05496 | C11H9O3 | -4.007 | 189.05499(100),147.04427(6),145.06468(5),121.06489(1) | Dimethyl-hydroxychromone or isomers |
| H52 | 5.18 | 405.11948 | C20H21O9 | 0.925 | 243.06613(100) | Glucosyl piceatannol |
| H53 | 5.26 | 503.12000 | C24H23O12 | 0.995 | 313.05676(55),189.05495(63),169.01331(100),151.00261(10),125.02313(20) | 2,5-Dimethyl-7-hydroxy-chromene-galloyl-glucose or isomers |
| H54 | 5.28 | 309.09814 | C15H17O7 | 0.530 | 189.05508(16),161.05978(74),147.04407(100) | 2-Cinnamoyl-glucose |
| H55 | 5.30 | 219.06581 | C12H11O4 | -2.155 | 219.06522(33),189.05489(77),161.05978(100),133.06483(74),131.04916(42) | 2-Methyl-5-carboxy methyl-7-hydroxy-chromene |
| H56 | 5.30 | 311.05640 | C17H11O6 | 0.928 | 293.04648(100),265.05142(48),255.06645(30) |  |
| H57 | 5.30 | 431.09860 | C21H19O10 | 0.534 | 293.04678(10),269.04642(100),240.04288(5) | Aloe emodin-glucoside |
| H58 | 5.42 | 477.14041 | C23H25O11 | 0.367 | 477.14142(21),313.05771(36),211.02483(34),169.01372(100),125.02346(20) | Isolindleyin |
| H59 | 5.46 | 355.10376 | C16H19O9 | 0.858 | 189.05486(14),161.05991(30),147.04398(100),113.02316(35) |  |
| H60 | 5.46 | 449.10950 | C21H21O11 | 1.662 | 287.05634(39),151.00262(100),135.04405(40) | Glucosyl eriodictyol |
| H61 | 5.46 | 503.12030 | C24H23O12 | 1.591 | 313.05679(24),189.05495(100),169.01331(18),151.00267(8) | 2,5-Dimethyl-7-hydroxy-chromene-galloyl-glucose or isomers |
| H62 | 5.46 | 581.22504 | C28H37O13 | 1.851 |  | Lyoniresinol-3a-O-β-glucoside or isomers |
| H63 | 5.49 | 479.12000 | C22H23O12 | 1.045 | 313.05685(63),169.01329(100),125.02316(21) |  |
| H64 | 5.50 | 235.06100 | C12H11O5 | -0.837 | 191.07069(100),149.02325(8) | 3-(4-Acetoxy-3-methoxyphenyl)acrylic acid |
| H65 | 5.56 | 607.13080 | C27H27O16 | 0.564 | 283.02512(100),239.03499(40) | Diglucosyl-rhein |
| H67* | 5.57 | 477.14142 | C23H25O11 | 2.484 | 331.06827(16),313.05765(28),169.01375(100) | Lindleyin |
| H68* | 5.57 | 609.14630 | C27H29O16 | 0.414 | 301.03552(47),300.02768(100),281.08179(9) | Rutin |
| H66 | 5.57 | 441.08328 | C22H17O10 | 1.270 | 289.07269(24),169.0137(100),125.02352(42) | Galloyl-catechin |
| H69 | 5.59 | 463.08920 | C21H19O12 | 2.161 | 301.03534(100),175.00264(4),151.00264(22) | Gentiopicroside isomers |
| H70 | 5.60 | 559.16809 | C24H31O15 | 2.230 | 193.05043(72),175.03960(100),160.01599(20),149.06007(16),134.03642(34) |  |
| H71 | 5.63 | 319.04614 | C15H11O8 | 0.625 | 231.06636(69),177.05486(44),168.00537(100),149.02298(31),109.02808(36) |  |
| H72 | 5.64 | 459.09396 | C22H19O11 | 1.471 | 415.10489(4),253.05122(100) |  |
| H73 | 5.65 | 503.12027 | C24H23O12 | 1.532 | 313.05643(31),189.05495(100),169.01295(39),125.02328(6) | 2,5-Dimethyl-7-hydroxy-chromene-galloyl-glucose or isomers |
| H74 | 5.69 | 395.13467 | C19H23O9 | -0.216 | 395.13596(38),233.08221(100),217.05081(17) | 2-(2'-Hydroxypropyl)-5-methyl-7-hydroxychromone 7-O-β-D-glucopyranoside |
| H75 | 5.69 | 417.11951 | C21H21O9 | 0.970 | 255.06696(100),213.05559(14) | Glucosyl pterostilbene |
| H76 | 5.72 | 231.06607 | C13H11O4 | -0.918 | 231.06586(100),189.05495(62),151.03896(12) | 2-Methyl-5-acetonyl-7-hydroxychromone or isomers |
| H77* | 5.77 | 463.08920 | C21H19O12 | 2.161 | 301.03470(6),300.02777(11),255.06624(100) | Gentiopicroside |
| H78 | 5.81 | 371.17154 | C18H27O8 | 1.075 | 371.17126(100),209.11790(22) | (2S)-4-[(4-Methoxybenzyl)oxy]-2-butanyl β-D-glucopyranoside or isomers |
| H79 | 5.86 | 623.16284 | C28H31O16 | 1.736 | 277.05078(11),266.05847(3),253.05066(100) |  |
| H80* | 5.90 | 243.06612 | C14H11O4 | -0.667 | 243.06677(20),163.03949(100),119.04926(42) | Piceatannol |
| H82* | 5.90 | 419.13513 | C21H23O9 | 0.894 | 257.08264(100),242.05830(51),241.05104(81) | Rhaponiticin |
| H81 | 5.90 | 371.17133 | C18H27O8 | 0.509 | 371.17148(100),209.11792(22) | 4-Hydroxy-3-(hydroxymethyl)-2-pentylphenyl D-glucopyranoside or isomers |
| H83 | 5.90 | 465.14056 | C22H25O11 | 0.699 | 257.08264(100),241.05099(18) |  |
| H84 | 5.92 | 233.08163 | C13H13O4 | -1.296 | 233.08231(73),189.05544(100) | 2-(2'-Hydroxypropyl)-5-methyl-7-hydroxychromone |
| H85 | 5.93 | 507.11404 | C23H23O13 | -0.737 | 235.06082(37),211.02411(38),193.0504(30),169.01326(100),151.00244(22),125.02322(34) |  |
| H86* | 5.98 | 847.21039 | C42H39O19 | 1.52 | 479.11325(32),389.09094(66),386.10129(100),253.05055(29),227.03450(57),224.04707(76) | Sennoside C |
| H87 | 6.03 | 583.11017 | C28H23O14 | 1.443 | 313.05734(51),269.04648(77),251.0359(23),169.01373(100) | Galloyl-emodin-glucoside |
| H88 | 6.08 | 609.14642 | C27H29O16 | 0.512 | 285.04080(100),257.04407(3) | Rheinoside B |
| H89 | 6.09 | 231.06615 | C13H11O4 | -0.572 | 231.06593(100),188.04695(29) | 2-Methyl-5-acetonyl-7-hydroxychromone or isomers |
| H90 | 6.12 | 433.11438 | C21H21O10 | 0.831 | 271.06219(100),151.00293(40) | Glucosyl naringenin |
| H91 | 6.15 | 371.17151 | C18H27O8 | 0.994 | 371.17148(100),209.11778(42),119.03354(22) | 4-Hydroxy-3-(hydroxymethyl)-2-pentylphenyl D-glucopyranoside or isomers |
| H92 | 6.18 | 517.09949 | C24H21O13 | 1.404 | 473.11023(14),311.05719(100),269.04718(4) |  |
| H93* | 6.24 | 447.09357 | C21H19O11 | 0.638 | 447.09442(3),284.03360(100), | Quercitrin |
| H94 | 6.26 | 165.05469 | C9H9O3 | -6.225 | 165.05515(100),149.02370(32),123.04423(36),121.06487(5) |  |
| H95* | 6.34 | 445.07797 | C21H17O11 | 0.754 | 307.02505(7),283.02573(75),239.03539(100) | Rhein-8-O-β-D-glucoside |
| H96* | 6.46 | 861.18805 | C42H37O20 | -0.368 | 449.10162(37),389.08862(54),386.10147(74),227.03517(62),224.04701(100) | Sennoside A |
| H97 | 6.48 | 577.15698 | C27H29O14 | 1.215 | 253.05124(100) | 5-Hydroxy-3-(4-hydroxyphenyl)-4-oxo-4H-chromen-7-yl 6-O-(6-deoxy-α-L-mannopyranosyl)-β-D-glucopyranoside or isomers |
| H98 | 6.60 | 461.10947 | C22H21O11 | 1.161 | 313.05692(38),211.02444(43),169.01334(100),151.00262(37),147.04408(80) | 2-O-Cinnamoyl-1-O-galloyl-β-D-glucopyranoside or isomers |
| H99 | 6.63 | 189.05492 | C11H9O3 | -4.218 | 189.05493(100),174.03085(1) | Dimethyl-hydroxychromone or isomers |
| H100 | 6.69 | 431.09848 | C21H19O10 | 0.255 | 431.09967(27),269.04648(100),240.04341(12) | Aloe emodin-glucoside |
| H101 | 6.72 | 593.15186 | C27H29O15 | 1.124 | 285.04089(12),269.04404(24),268.03802(66) | Diglucosyl-emodin |
| H102 | 6.73 | 209.11774 | C12H17O3 | -2.763 |  |  |
| H103 | 6.73 | 475.08865 | C22H19O12 | 0.949 | 283.06091(9),269.04651(100) | 6-(Hexopyranosyloxy)-3,8-dihydroxy-1-methyl-9,10-dioxo-9,10-dihydro-2-anthracenecarboxylic acid or isomers |
| H104 | 6.73 | 577.15704 | C27H29O14 | 1.319 | 253.05125(100) | 5-Hydroxy-3-(4-hydroxyphenyl)-4-oxo-4H-chromen-7-yl 6-O-(6-deoxy-α-L-mannopyranosyl)-β-D-glucopyranoside or isomers |
| H105 | 6.78 | 487.08899 | C23H19O12 | 1.624 | 305.04648(9),281.04578(80),253.05054(100) | Methyl (6aR,7S,10aR)-5,6a,7,10a,12-pentahydroxy-3,8-dimethoxy-1-methyl-6,10,11-trioxo-6,6a,7,10,10a,11-hexahydro-2-tetracenecarboxylate |
| H106 | 6.79 | 473.10947 | C23H21O11 | 1.132 | 293.04587(11),269.04584(100) | Acetyl glucosyl-aloe emodin |
| H107 | 6.86 | 475.08847 | C22H19O12 | 0.570 | 431.09991(80),269.04535(28),268.03854(100) | 6-(Hexopyranosyloxy)-3,8-dihydroxy-1-methyl-9,10-dioxo-9,10-dihydro-2-anthracenecarboxylic acid or isomers |
| H108 | 6.99 | 445.07809 | C21H17O11 | 1.023 | 283.06216(12),269.0464(100),239.07205(14) | Glucosyl rhein |
| H109 | 7.00 | 312.12445 | C18H18O4N | 1.021 | 312.12506(28),297.10144(23),178.05057(67),148.05223(100) |  |
| H110 | 7.05 | 607.16815 | C28H31O15 | 2.152 | 283.06131(100),240.04242(5) | Diglucosyl-physcion |
| H111 | 7.08 | 627.09985 | C29H23O16 | 1.104 | 313.05765(22),269.04645(100),169.01372(45),125.02354(8) |  |
| H112 | 7.15 | 342.13489 | C19H20O5N | 0.567 | 342.13565(21),327.11209(28),190.05069(24),178.05058(90),148.05223(100),135.04422(26) |  |
| H113 | 7.17 | 417.11948 | C21H21O9 | 0.898 | 255.06328(14),254.05908(100) | Glucosyl pterostilbene isomers |
| H114 | 7.18 | 461.10947 | C22H21O11 | 1.161 | 169.01331(100),161.05981(28),151.00259(41),125.02316(35) | 2-O-Cinnamoyl-1-O-galloyl-β-D-glucopyranoside or isomers |
| H115 | 7.20 | 577.15710 | C27H29O14 | 1.423 | 253.05136(100) | 5-Hydroxy-3-(4-hydroxyphenyl)-4-oxo-4H-chromen-7-yl 6-O-(6-deoxy-α-L-mannopyranosyl)-β-D-glucopyranoside or isomers |
| H116 | 7.24 | 275.05646 | C14H11O6 | 1.268 | 231.06653(100),191.03477(24),188.04767(30) | 2,5-Dimethyl-7-hydroxy chromone or isomers |
| H117 | 7.31 | 607.16791 | C28H31O15 | 1.757 | 283.06137(100),240.04253(10) | Diglucosyl-physcion |
| H118 | 7.33 | 593.15179 | C27H29O15 | 1.006 | 282.05280(1),269.04321(20),268.03790(100),253.04990(1) | Diglucosyl-emodin |
| H119 | 7.34 | 861.18768 | C42H37O20 | -0.797 |  | Sennoside B |
| H120 | 7.35 | 613.12042 | C29H25O15 | 0.859 | 465.06714(27),313.05701(43),271.04626(27),253.05038(23),211.02432(27),169.01331(100) | Di-galloyl-O-cinnamoyl-β-D-glucose or isomers |
| H121 | 7.36 | 287.05624 | C15H11O6 | 0.448 | 151.00299(98),135.0443(100),125.02351(23) | Eriodictyol |
| H122 | 7.36 | 459.09375 | C22H19O11 | 1.014 | 266.0592(29),253.05122(100) |  |
| H123 | 7.37 | 299.05621 | C16H11O6 | 0.330 | 299.05692(1),255.06621(100) | Fallacinol |
| H124 | 7.44 | 407.13492 | C20H23O9 | 0.404 | 245.08244(100),230.05867(20) | 7-Acetyl-8-hydroxy-3-methoxy-6-methyl-1-naphthyl β-D-allopyranoside |
| H125 | 7.45 | 325.07178 | C18H13O6 | 0.057 | 307.0618(19),289.05103(45),261.05566(39),253.05067(100) | Lactylchrysophanol |
| H126 | 7.45 | 397.09378 | C21H17O8 | 2.239 | 291.06555(2),253.05069(100) |  |
| H127 | 7.46 | 415.10370 | C21H19O9 | 0.589 | 277.05136(10),253.05124(100) | Chrysophanol 1-O-glucoside |
| H128 | 7.46 | 431.09854 | C21H19O10 | 0.394 | 431.09949(1),269.04385(13),268.03851(100),253.04950(1) | Emodin-glucoside |
| H129 | 7.46 | 847.21014 | C42H39O19 | 1.225 | 431.09851(2),269.04156(5),268.03781(100),253.05077(7) | Sennoside D |
| H130 | 7.49 | 403.14008 | C21H23O8 | 0.593 | 241.08678(100),225.0553(18) | Desoxyrhaponticin |
| H131 | 7.51 | 449.14563 | C22H25O10 | 0.690 | 241.08684(100),225.05519(6) | Rumexneposides B isomers |
| H132 | 7.52 | 275.05640 | C14H11O6 | 1.049 | 275.05704(56),231.06668(100),203.07132(36) | 2,5-Dimethyl-7-hydroxy chromone or isomers |
| H133 | 7.57 | 473.10913 | C23H21O11 | 0.413 | 473.10962(14),311.05643(100),253.05067(13) | Acetyl glucosyl-aloe emodin |
| H134 | 7.59 | 549.03448 | C23H17O14S | 0.056 | 269.04639(10),169.01405(3),116.92753(100) | Sulfated acetyl glucosyl aloe emodin |
| H136* | 7.61 | 431.09842 | C21H19O10 | 0.116 | 431.09991(11),269.04642(100),240.04240(1),225.05675(1) | Emodin-8-O-β-D-glucoside |
| H135 | 7.61 | 325.07205 | C18H13O6 | 0.888 | 307.06210(21),289.05075(56),261.05600(55),253.05069(100) | Lactylchrysophanol |
| H137 | 7.64 | 397.09314 | C21H17O8 | 0.628 | 291.06699(3),253.05063(100),239.07042(6) |  |
| H138 | 7.64 | 415.10376 | C21H19O9 | 0.734 | 277.05151(10),253.05128(100) | Chrysophanol 8-O-glucoside |
| H139 | 7.66 | 285.04059 | C15H9O6 | 0.452 | 285.04141(100),257.0462(2),269.04453(1),256.03806(3),241.05092(1) | ω-Hydroxy-emodin |
| H140* | 7.73 | 301.03555 | C15H9O7 | 0.578 | 301.03558(43),178.99823(42),151.00296(100) | Quercetin |
| H141 | 7.75 | 317.03030 | C15H9O8 | 0.030 | 299.01981(79),273.03998(67),153.01842(61),151.00212(57),109.02851(100) |  |
| H142 | 7.77 | 613.12036 | C29H25O15 | 0.761 | 401.08817(13),313.05682(17),271.04623(45),211.02426(30),169.01329(100),125.02313(21) | Di-galloyl-O-cinnamoyl-β-D-glucose or isomers |
| H143 | 7.80 | 355.04636 | C18H11O8 | 1.181 | 311.05722(100),269.04651(19),240.04295 | Related to H201 |
| H144 | 7.82 | 607.16797 | C28H31O15 | 1.856 | 283.06140(100),240.04234(8) | Diglucosyl-physcion |
| H145 | 7.85 | 499.12570 | C25H23O11 | 2.235 | 293.04581(12),269.04575(100),240.04237(7) | 4,5-Dihydroxy-7-methyl-9,10-dioxo-9,10-dihydro-2-anthracenyl 3,4-di-O-acetyl-6-deoxyhexopyranoside or isomers |
| H146 | 7.86 | 487.08878 | C23H19O12 | 1.192 | 307.02539(4),283.02518(43),263.03436(5),239.03485(100) | Acetyl glucosyl-rhein |
| H147 | 7.86 | 569.13062 | C28H25O13 | 0.977 | 313.05783(6),255.06723(12),169.01379(18),125.02349(100) | 2″-O-galloylvitexin |
| H148 | 7.86 | 613.12054 | C29H25O15 | 1.055 | 401.08820(8),271.04630(6),211.02420(10),169.01331(100) | Di-galloyl-O-cinnamoyl-β-D-glucose or isomers |
| H149 | 7.88 | 567.11438 | C28H23O13 | -0.060 | 313.05804(34),275.02048(16),253.05188(36),169.01373(100) | Galloyl-chrysophanol-1-O-glucoside |
| H150 | 8.02 | 621.12573 | C31H25O14 | 1.210 | 311.05673(12),282.05350(13),269.04578(100) |  |
| H151 | 8.10 | 325.07196 | C18H13O6 | 0.611 | 292.03793(100),281.04575(74),253.05058(17) | 2-[(5-Hydroxy-4-oxo-2-phenyl-4H-chromen-7-yl)oxy]propanoic acid or isomers |
| H152 | 8.11 | 445.11450 | C22H21O10 | 1.078 | 283.06137(100) | Glucosyl physcion |
| H153 | 8.12 | 567.11511 | C28H23O13 | 1.227 | 313.05762(37),253.05150(45),169.01373(100),125.02357(18) | Galloyl-chrysophanol-8-O-glucoside |
| H154 | 8.15 | 401.12451 | C21H21O8 | 0.796 | 281.08279(100),253.08733(3),239.07159(3) |  |
| H155 | 8.22 | 299.05624 | C16H11O6 | 0.430 | 299.05582(34),255.06624(100),231.06618(21) |  |
| H156 | 8.23 | 217.05025 | C12H9O4 | -1.760 | 217.05020(100),191.03421(35),175.03934(31) | Dimethyl-hydroxy-acetyl-chromene or isomers |
| H157 | 8.25 | 209.11781 | C12H17O3 | -2.428 | 209.11835(93),191.10747(100),165.12794(35),135.08067(45) |  |
| H158 | 8.30 | 623.17712 | C32H31O13 | 0.170 | 169.01338(100),163.03926(80),151.00281(18),145.02811(38),125.02324(37) |  |
| H159 | 8.33 | 325.07187 | C18H13O6 | 0.334 | 292.03809(100),281.04587(95),264.04398(25),236.04878(21) | 2-[(5-Hydroxy-4-oxo-2-phenyl-4H-chromen-7-yl)oxy]propanoic acid or isomers |
| H160 | 8.34 | 271.06137 | C15H11O5 | 0.639 | 271.06235(32),177.01918(11),151.00301(100),119.04925(57) | Naringenin |
| H161 | 8.34 | 297.07678 | C17H13O5 | -0.225 | 281.04572(26),253.05058(100) | 1,8-Dihydroxy-3-methoxy-2,6-dimethylanthracene-9,10-dione or isomers |
| H162 | 8.34 | 445.11435 | C22H21O10 | 0.741 | 283.06134(100) | Glucosyl physcion |
| H163 | 8.42 | 517.09937 | C24H21O13 | 1.172 | 473.11063(29),269.04639(100),268.03860(38),240.04286(1) |  |
| H164 | 8.50 | 217.05017 | C12H9O4 | -2.129 | 217.05022(100),199.03943(21),175.03944(16) | Dimethyl-hydroxy-acetyl-chromene or isomers |
| H165 | 8.58 | 635.14075 | C32H27O14 | 0.191 | 268.03818(9),253.05057(100) |  |
| H166 | 8.67 | 473.10931 | C23H21O11 | 0.793 | 473.10861(7),311.05594(3),269.04556(100),225.05528(2) | Acetyl glucosyl-emodin |
| H167 | 8.70 | 317.14301 | C15H25O5S | 0.606 | 317.14383(66),273.04187(1),96.95896(100) |  |
| H168 | 8.73 | 313.03574 | C16H9O7 | 1.163 | 295.06192(7),269.04581(100).254.05855(51),253.05038(23) | Laccaic acid |
| H169 | 8.79 | 605.13068 | C31H25O13 | 1.018 | 253.05069(100) |  |
| H170* | 8.83 | 285.04059 | C15H9O6 | 0.452 | 285.04141(100),257.0462(2),229.05086(2) | Kaempferol |
| H171 | 8.83 | 301.03546 | C15H9O7 | 0.279 | 245.04613(100),206.99348(23),151.00296(65) |  |
| H172 | 8.83 | 473.10928 | C23H21O11 | 0.730 | 473.10962(1),269.04388(28),268.03793(100) | Acetyl glucosyl-aloe emodin |
| H173 | 8.95 | 449.14575 | C22H25O10 | 0.957 | 245.08183(100),230.05817(25) | Rumexneposides B |
| H174 | 9.01 | 621.12592 | C31H25O14 | 1.516 | 282.05392(1),269.04572(100) |  |
| H175 | 9.02 | 699.13519 | C36H27O15 | -0.505 | 475.08182(100),283.02536(71),269.04529(35),253.05119(57),239.03404(32) | 5-(β-D-Glucopyranosyloxy)-4,4',5'-trihydroxy-10,10'-dioxo-9,9',10,10'-tetrahydro-9,9'-bianthracene-2,2'-dicarboxylic acid or isomers |
| H176 | 9.06 | 457.11450 | C23H21O10 | 1.050 | 277.05078(11),253.05061(100),239.07127(4) | Acetyl glucosyl-chrysophanol |
| H177 | 9.08 | 317.14294 | C15H25O5S | 0.385 | 317.14380(70),96.95896(100) |  |
| H178 | 9.14 | 699.13586 | C36H27O15 | 0.453 | 519.07361(38),475.08295(99),283.0246(100),269.04626(29),253.0506(29) | 5-(β-D-Glucopyranosyloxy)-4,4',5'-trihydroxy-10,10'-dioxo-9,9',10,10'-tetrahydro-9,9'-bianthracene-2,2'-dicarboxylic acid or isomers |
| H179 | 9.16 | 457.11438 | C23H21O10 | 0.787 | 277.0509(12),253.0506(100),239.07083(4) | Acetyl glucosyl-chrysophanol |
| H180 | 9.28 | 299.01993 | C15H7O7 | 0.683 | 299.02078(41),284.03281(9),255.02989(100),227.03505(11) | Laccaic acid D or isomers |
| H181 | 9.31 | 621.12604 | C31H25O14 | 1.709 | 269.04587(100) |  |
| H182 | 9.38 | 313.03577 | C16H9O7 | 1.259 | 313.03513(21),269.04556(100) | 4,5-Dihydroxy-7-methoxy-9,10-dioxo-9,10-dihydro-2-anthracenecarboxylic acid |
| H183 | 9.51 | 325.07169 | C18H13O6 | -0.220 | 281.08203(51),266.05853(92),265.05057(100),238.06345(77) |  |
| H184* | 9.52 | 269.04562 | C15H9O5 | 0.273 | 269.04651(100),240.04318(27) | Aloe emodin |
| H185 | 9.52 | 297.07697 | C17H13O5 | 0.415 | 297.07681(6),282.05347(100),253.05075(8) | 1,8-Dihydroxy-3-methoxy-2,6-dimethylanthracene-9,10-dione or isomers |
| H186 | 9.55 | 633.16217 | C33H29O13 | 1.273 | 189.05547(100),169.01376(82),151.00304(20),125.02354(30) |  |
| H187 | 9.60 | 313.03568 | C16H9O7 | 0.972 | 313.03464(1),269.04581(100) | Endocrocin |
| H188 | 9.60 | 591.15149 | C31H27O12 | 1.168 | 299.05627(100),281.04614(4),255.06631(76),231.06546(7) |  |
| H189 | 9.74 | 487.12527 | C24H23O11 | 1.407 | 283.06134(100),268.03757(4),240.04231(17) | Acetyl glucosyl-physcion |
| H190 | 9.75 | 699.13623 | C36H27O15 | 0.982 | 475.08289(37),457.073(19),283.02536(100),253.05002(52),239.03465(37) | 5-(β-D-Glucopyranosyloxy)-4,4',5'-trihydroxy-10,10'-dioxo-9,9',10,10'-tetrahydro-9,9'-bianthracene-2,2'-dicarboxylic acid or isomers |
| H191 | 9.82 | 355.04626 | C18H11O8 | 0.900 | 311.05737(100),269.04413(3) | Related to H201 |
| H192 | 9.85 | 605.13086 | C31H25O13 | 1.316 | 311.05533(3),282.05362(34),269.04584(100) |  |
| H193 | 9.90 | 487.12515 | C24H23O11 | 1.161 | 283.06140(100),268.03793(4),240.04251(19) | Acetyl glucosyl-physcion |
| H194 | 9.90 | 523.10187 | C34H19O4S | 1.753 | 283.06128(100),240.04381(4),152.99469(15) |  |
| H195 | 9.90 | 607.18268 | C32H31O12 | 0.956 | 283.05807(38),269.046330(100),169.01366(15) |  |
| H196 | 9.93 | 287.05634 | C15H11O6 | 0.797 | 243.06683(100),225.05597(7),163.00316(23),123.04427(23) | 3,5-Dihydroxy-2-[2-(4-hydroxyphenyl) acetyl]benzoic acid |
| H197 | 9.94 | 633.16205 | C33H29O13 | 1.083 | 189.05545(100),169.01376(13) |  |
| H198 | 9.96 | 499.12518 | C25H23O11 | 1.193 | 268.03790(100) | 4,5-Dihydroxy-7-methyl-9,10-dioxo-9,10-dihydro-2-anthracenyl 3,4-di-O-acetyl-6-deoxyhexopyranoside or isomers |
| H199 | 10.16 | 317.14301 | C15H25O5S | 0.606 | 317.14395(51),258.05414(17),96.95895(100) |  |
| H200 | 10.32 | 607.18280 | C32H31O12 | 1.154 | 254.05865(29),169.01392(100),151.00322(34),125.02356(35) |  |
| H201 | 10.33 | 311.05643 | C17H11O6 | 1.024 | 311.05725(100),296.06863(50),268.03860(26) | Acetylaloe emodin |
| H202 | 10.53 | 299.05630 | C16H11O6 | 0.631 | 299.05627(72),255.06615(55),240.04266(100) | Oxidized physcion |
| H203 | 10.58 | 669.16254 | C36H29O13 | 1.757 | 507.10950(4),489.10065(14),471.08862(3),461.10556(5),253.05074(100) | (1ξ)-1,5-Anhydro-1-[(10'S)-1',4,5,8',10'-pentahydroxy-2,6'-dimethyl-9,9',10-trioxo-9,9',10,10'-tetrahydro-1,2'-bianthracen-10'-yl]-D-glucitol or isomers |
| H204 | 10.67 | 303.08768 | C16H15O6 | 0.886 | 259.09744(52),244.07413(100) | Methylcatechin |
| H205 | 10.68 | 255.06644 | C15H11O4 | 0.619 | 255.06325(55), | Pterostilbene or isomers |
| H206 | 10.78 | 299.01993 | C15H7O7 | 0.683 | 299.02011(100),284.03287(7),255.02975(43),240.04346(21) | Laccaic acid D or isomers |
| H207 | 11.27 | 605.13080 | C31H25O13 | 1.216 | 561.14026(54),269.04486(26),268.03772(100),240.0421(3) |  |
| H208 | 11.29 | 589.13562 | C31H25O12 | 0.799 | 266.05933(41),253.05127(100) |  |
| H209 | 11.31 | 396.96558 | C18H5O9S | -0.997 | 353.97870(13),352.97723(47),295.02563(23),116.92754(100) |  |
| H210 | 11.43 | 605.13086 | C31H25O13 | 1.316 | 561.14056(34),269.04391(24),268.03784(100),240.04245(4) |  |
| H211* | 11.58 | 283.02505 | C15H7O6 | 0.844 | 283.02579(12),257.04623(60),239.03545(100),183.04471(19) | Rhein |
| H212 | 12.10 | 669.16241 | C36H29O13 | 1.563 | 489.0983(17),471.0889(4),268.03851(9),253.05058(100) | (1ξ)-1,5-Anhydro-1-[(10'S)-1',4,5,8',10'-pentahydroxy-2,6'-dimethyl-9,9',10-trioxo-9,9',10,10'-tetrahydro-1,2'-bianthracen-10'-yl]-D-glucitol or isomers |
| H213 | 12.42 | 311.05640 | C17H11O6 | 0.928 | 311.05731(100),283.06229(2),240.04260(2) | 1,3,6,8-Tetrahydroxy-2-methyl-7-vinyl-9,10-anthraquinone |
| H214 | 13.07 | 299.02005 | C15H7O7 | 1.084 |  | Laccaic acid D or isomers |
| H215* | 13.16 | 269.04565 | C15H9O5 | 0.384 | 269.04648(100),241.05118(2),225.05609(5) | Emodin |
| H216 | 14.69 | 255.06746 | C15H11O4 | 4.618 | 255.06502(100),213.05533(12),151.00247(8) | Pterostilbene or isomers |
| H217* | 14.70 | 253.05066 | C15H9O4 | 0.110 | 253.05133(100),225.05586(6) | Chrysophanol |
| H218 | 15.07 | 387.12427 | C24H19O5 | 1.222 | 240.07521(10),239.07184(100) |  |
| H219 | 15.77 | 523.10413 | C30H19O9 | 1.290 | 505.09387(7),269.04617(37),253.05051(50),240.04233(17),152.99481(100) | Sennidin C |
| H220* | 16.26 | 283.06137 | C16H11O5 | 0.612 | 283.06216(98),268.03836(8),240.0433(100) | Physcion |
| H221 | 17.41 | 537.08313 | C30H17O10 | 0.764 | 475.08496(100),283.02612(83),255.23424,253.05128(90),239.03589(38) |  |
| H222 | 19.40 | 405.12280 | C17H25O9S | 0.799 | 387.11206(46),361.13351(100), |  |
| H223 | 19.69 | 507.10889 | C30H19O8 | 0.689 | 489.09738(7),279.23297(36),253.05072(100) | Rheidin B |

*Compounds validated by reference substances

**Supplementary Table 2.** Related components of rhubarb *in vivo*

| No. | RT | HRMS- | Formula | Error(ppm) | ms2 | Compounds | M/R | Human |
| --- | --- | --- | --- | --- | --- | --- | --- | --- |
| H1 | 0.58 | 331.06732 | C13H15O10 | 0.756 | 331.06821(11),211.02481(28),169.0137(0100),125.02350(18) | Glucosyl-gallic acid | + |  |
| H3 | 0.90 | 331.06726 | C13H15O10 | 0.574 | 331.06842(8),169.01373(100),125.02348(19) | Glucosyl-gallic acid | + |  |
| H4* | 1.05 | 169.01332 | C7H5O5 | -5.482 | 169.01302(43),125.02295(100) | Gallic acid | + | + |
| MR1 | 1.13 | 507.09955 | C19H23O16 | 0.774 | 331.06750(45),169.01340(100) | Glucuronidated glucosyl-gallic acid | + |  |
| H6 | 1.33 | 345.08301 | C14H17O10 | 1.387 | 256.01514(100),255.00749(78),245.99448(81) | Methyl-glucosyl-gallic acid | + |  |
| H9 | 1.67 | 331.06735 | C13H15O10 | 0.846 | 169.01370(100),125.02345(25) | Glucosyl-gallic acid | + |  |
| MP1 | 1.76 | 317.05115 | C12H13O10 | -0.851 | 141.01822(84),123.00761(33),113.02317(100) |  | + | + |
| MP2 | 1.84 | 359.06186 | C14H15O11 | -0.347 | 183.02911(48),168.00551(100),113.02316(41) | Glucuronidated methylgallic acid | + | + |
| MP3 | 2.29 | 359.06201 | C14H15O11 | 0.071 | 183.02908(100),168.00551(83),139.03891(33) | Glucuronidated methylgallic acid | + | + |
| H11 | 2.61 | 183.02911 | C8H7O5 | -4.298 | 183.02945(29),168.00552(100),124.01543(64) | Methylgallic acid | + |  |
| H12 | 2.63 | 219.05072 | C8H11O7 | -1.397 | 111.00749(100) | Dimethy citric acid | + |  |
| MP4 | 2.69 | 359.06189 | C14H15O11 | -0.263 | 183.02917(100),168.00554(37),139.03891(13) | Glucuronidated methylgallic acid | + | + |
| H14 | 2.78 | 183.02911 | C8H7O5 | -4.298 | 183.02917(4),169.00905(7),168.00554(100),125.01865(2),124.01539(49) | Methylgallic acid | + |  |
| MR2 | 3.20 | 339.07254 | C15H15O9 | 1.135 | 163.03912(100) | Glucuronidated coumaric acid | + |  |
| MR3 | 3.49 | 465.10440 | C21H21O12 | 1.184 | 289.07196(100),245.0818(46) | Glucuronidated catechin | + |  |
| MR4 | 3.60 | 409.07819 | C18H17O11 | 1.358 | 189.05508(100),113.02318(23) |  | + |  |
| H29 | 3.77 | 165.05478 | C9H9O3 | -5.680 | 165.05508(100),149.02365(11),123.04416(25),121.06487(71) |  | + |  |
| MP5 | 3.88 | 373.07745 | C15H17O11 | -0.495 | 197.04506(74),182.02135(100),113.02317(53) | Glucuronidated dimethylgallic acid | + | + |
| MR5 | 4.17 | 339.07251 | C15H15O9 | 1.046 | 193.03506(38),163.03902(56),113.02307(100) | Glucuronidated coumaric acid | + |  |
| MP6 | 4.32 | 409.11444 | C19H21O10 | 1.027 | 233.08173(100),189.05504(29),113.02312(41) |  | + | + |
| MP7 | 4.60 | 373.07739 | C15H17O11 | -0.655 | 198.04829(10),197.04491(100) | Glucuronidated dimethylgallic acid | + | + |
| MR6 | 4.85 | 607.13080 | C27H27O16 | 0.564 | 269.04532(63),268.03806(100) | Glucuronidated glucosyl emodin | + |  |
| H44 | 4.86 | 197.04465 | C9H9O5 | -4.551 | 197.04509(100),182.02132(35),169.01332(45),153.05473(76),138.03127(39),129.05464(51) | Dimethylgallic acid | + |  |
| MP8 | 5.00 | 387.09320 | C16H19O11 | -0.218 | 211.06128(23),196.03711(100) | Glucuronidated trimethylgallic acid | + | + |
| MR7 | 5.11 | 339.07251 | C15H15O9 | 1.046 | 163.03925(66),119.04905(100),113.02320(31) | Glucuronidated coumaric acid | + |  |
| H51 | 5.17 | 189.05496 | C11H9O3 | -4.007 | 189.05499(100),147.04427(6),145.06468(5),121.06489(1) | Dimethyl-hydroxychromone or isomers | + |  |
| MR8 | 5.32 | 347.07901 | C14H19O8S | -4.614 | 217.01579(70),200.14000(27),164.07072(10) |  | + |  |
| MR9 | 5.33 | 435.09396 | C20H19O11 | 1.552 | 215.07101(100) |  | + |  |
| MR10 | 5.40 | 315.05292 | C16H11O7 | 6.012 | 315.05411(11),111.00752(100) |  | + |  |
| MR11 | 5.46 | 347.07904 | C14H19O8S | -4.528 | 217.01570(69),200.13943(20),155.01463(18),111.00748(100) |  | + |  |
| H64 | 5.50 | 235.06100 | C12H11O5 | -0.837 | 191.07069(100),149.02325(8) | 3-(4-Acetoxy-3-methoxyphenyl) acrylic acid | + |  |
| MR12 | 5.53 | 343.10400 | C15H19O9 | 3.186 | 167.07057(53),152.04684(91),113.02310(69) |  | + |  |
| MP9 | 5.56 | 445.07751 | C21H17O11 | -0.280 | 269.04590(100),240.04248(7) | Glucuronidated aloe-emodin | + | + |
| MP10 | 5.63 | 465.10458 | C21H21O12 | 1.571 | 271.06140(48),253.05090(76),113.02316(100) |  | + | + |
| MP11 | 5.63 | 387.09348 | C16H19O11 | 1.900 | 211.06050(18),196.03697(100),113.02320(53) |  | + | + |
| MP12 | 5.64 | 437.07318 | C19H17O12 | 1.443 | 261.04071(100),217.05058(37) |  | + | + |
| MR13 | 5.69 | 269.04517 | C15H9O5 | -1.400 | 269.04590(100),241.04750(6),240.04271(30) |  | + |  |
| H76 | 5.72 | 231.06607 | C13H11O4 | -0.918 | 231.06586(100),189.05495(62),151.03896(12) | 2-Methyl-5-acetonyl-7-hydroxychromone or isomers | + |  |
| MP13 | 5.80 | 373.07764 | C15H17O11 | 0.015 | 198.04831(7),197.04495(100),169.01308(7) | Glucuronidated dimethylgallic acid | + | + |
| MR14 | 5.80 | 607.13080 | C27H27O16 | 0.564 |  | Glucuronidated glucosyl emodin | + |  |
| H78 | 5.81 | 371.17154 | C18H27O8 | 1.075 | 371.17126(100),209.11790(22) | (2S)-4-[(4-Methoxybenzyl)oxy]-2-butanyl β-D-glucopyranoside or isomers | + |  |
| MR15 | 5.84 | 431.09836 | C21H19O10 | -0.023 | 255.06645(100) |  | + |  |
| H84 | 5.92 | 233.08163 | C13H13O4 | -1.296 | 233.08231(73),189.05544(100) | 2-(2'-Hydroxypropyl)-5-methyl-7-hydroxychromone | + |  |
| H89 | 6.09 | 231.06615 | C13H11O4 | -0.572 | 231.06593(100),188.04695(29) | 2-Methyl-5-acetonyl-7-hydroxychromone or isomers | + |  |
| MR16 | 6.09 | 409.11484 | C19H21O10 | 2.004 | 233.08171(100),191.07025(10) |  | + |  |
| MP14 | 6.10 | 431.09836 | C21H19O10 | -0.023 | 255.06636(100) |  | + | + |
| MR17 | 6.11 | 301.03583 | C15H9O7 | 1.509 | 257.04581(100),239.03499(69),229.05043(36),213.05556(38) | Viscidulin I | + |  |
| MP15 | 6.13 | 591.13550 | C27H27O15 | -0.073 | 253.05064(100) | Glucuronidated glucosyl chrysophanol | + | + |
| MP16 | 6.19 | 413.10907 | C18H21O11 | 0.328 | 237.07666(68),191.10695(40),85.02802(100) |  | + | + |
| MP17 | 6.21 | 323.07773 | C15H15O8 | 1.515 | 147.04407(40),113.02319(100) |  | + | + |
| MR18 | 6.23 | 287.05646 | C15H11O6 | 1.215 | 243.06635(8),225.05544(43),177.01854(37),109.02821(100) | Desaturated catechin | + |  |
| MP18 | 6.28 | 211.06062 | C10H11O5 | -2.733 | 196.03703(15),153.05482(100),152.04671(25) | Trimethylgllagic acid | + | + |
| MP19 | 6.31 | 391.10410 | C19H19O9 | 1.648 | 215.07100(100),113.02306(33) |  | + | + |
| MR19 | 6.32 | 461.07260 | C21H17O12 | 0.110 | 285.04086(100) | Glucuronidated oxidized emodin | + |  |
| MR20 | 6.32 | 285.04016 | C15H9O6 | -1.057 | 285.0408(100),241.05075(3) | Oxidized emodin | + |  |
| MR21 | 6.34 | 451.08896 | C20H19O12 | 1.687 | 275.05673(100),260.03262(69),216.04190(73),113.02296(33) |  | + |  |
| MR22 | 6.38 | 343.10339 | C15H19O9 | -0.191 | 256.01587(30),163.06033(100),101.02309(80) |  | + |  |
| MR23 | 6.41 | 445.07755 | C21H17O11 | -0.190 | 269.04590(100),240.04263(7) | Glucuronidated aloe-emodin | + |  |
| MR24 | 6.48 | 357.11969 | C16H21O9 | 3.173 | 181.08632(71),166.06253(92),113.02322(76),85.02803(100) |  | + |  |
| MP20 | 6.54 | 525.03394 | C21H17O14S | -0.969 |  | Glucuronidated sulfated emodin | + | + |
| MR25 | 6.59 | 473.10965 | C23H21O11 | 1.512 | 297.07709(100),253.05064(27),113.02318(11) |  | + |  |
| MP21 | 6.60 | 459.05719 | C21H15O12 | 0.634 | 283.02524(100),239.03481(44) | Glucuronidated rhein | + | + |
| MR26 | 6.61 | 461.07211 | C21H17O12 | -0.952 | 285.03946(92) | Glucuronidated oxidized aloe emodin | + |  |
| MR27 | 6.65 | 477.10370 | C22H21O12 | -0.313 | 301.07175(100),301.03537(63),151.00258(59),113.02313(32) |  | + |  |
| MR28 | 6.70 | 621.11066 | C27H25O17 | 1.510 |  | Diglucuronidated emodin | + |  |
| H102 | 6.73 | 209.11774 | C12H17O3 | -2.763 |  |  | + |  |
| MR29 | 6.74 | 249.07663 | C13H13O5 | -0.870 | 205.08710(71) |  | + |  |
| MR30 | 6.74 | 435.09390 | C20H19O11 | 1.414 | 259.06149(100),244.03749(45),231.06639(14) |  | + |  |
| MP22 | 6.76 | 621.14636 | C28H29O16 | 0.406 | 283.06146(100),240.04279(4) | Glucuronidated glucosyl physcion | + | + |
| MR31 | 6.78 | 437.07349 | C19H17O12 | 2.153 | 261.04083(100),217.05020(42),189.05484(10) |  | + |  |
| MR32 | 6.85 | 507.11496 | C23H23O13 | 1.077 | 316.02310(58),168.00548(86),166.99777(83),123.00755(96),113.02314(100) |  | + |  |
| MP23 | 6.90 | 459.05698 | C21H15O12 | 0.176 | 283.02493(18),257.04596(100),239.03481(48) | Glucuronidated rhein | + | + |
| MR33 | 6.90 | 525.03418 | C21H17O14S | -0.512 | 269.04553(100) | Glucuronidated sulfated emodin | + |  |
| MR34 | 6.93 | 605.11554 | C27H25O16 | 1.210 |  | Diglucuronidated chrysophanol | + |  |
| MR35 | 6.96 | 489.10358 | C23H21O12 | -0.550 | 313.07224(100),298.04846(10) |  | + |  |
| MR36 | 7.02 | 453.17664 | C22H29O10 | 0.044 | 233.15443(100),232.14685(51) |  | + |  |
| MR37 | 7.11 | 285.04037 | C15H9O6 | -0.320 | 285.04047(11),241.05054(100) |  | + |  |
| MR38 | 7.16 | 275.05658 | C14H11O6 | 1.704 | 275.05716(18),229.05034(42),217.05032(100) |  | + |  |
| MR39 | 7.18 | 315.05161 | C16H11O7 | 1.854 | 271.06168(83),256.03778(100),227.15514(41) |  | + |  |
| MR40 | 7.22 | 437.10956 | C20H21O11 | 1.431 | 261.07700(100),246.05331(40) |  | + |  |
| MR41 | 7.29 | 489.10388 | C23H21O12 | 0.063 | 313.07230(100) |  | + |  |
| MR42 | 7.34 | 473.10968 | C23H21O11 | 1.575 | 297.07755(48),283.06000(33),282.05374(100),113.02315(44) |  | + |  |
| MP24 | 7.35 | 633.14630 | C29H29O16 | 0.303 | 253.05080(100) | Glucuronidated acetyl glucosylchrysophanol | + | + |
| H123 | 7.37 | 299.05621 | C16H11O6 | 0.330 | 299.05692(1),255.06621(100) | Fallacinol | + |  |
| MR43 | 7.40 | 433.11380 | C21H21O10 | -0.508 | 257.08218(100) |  | + |  |
| MP25 | 7.40 | 188.07069 | C11H10O2N | -5.380 | 188.07069(23),144.04454(15),142.06508(10) |  | + | + |
| MP26 | 7.40 | 221.08121 | C12H13O4 | 6.500 | 221.08165(68),177.09132(32),161.05986(55),149.05977(92) |  | + | + |
| H127 | 7.46 | 415.10370 | C21H19O9 | 0.589 | 277.05136(10),253.05124(100) | Chrysophanol 1-O-glucoside | + | + |
| MR44 | 7.49 | 365.23303 | C21H33O5 | -0.869 | 365.23364(100),321.24420(5),303.23334(11) |  | + |  |
| MR45 | 7.49 | 349.00223 | C15H9O8S | -0.376 | 269.04584(100),240.04259(6) | Sulfated aloe emodin | + |  |
| MR46 | 7.49 | 487.08813 | C23H19O12 | -0.142 | 311.05652(100) |  | + |  |
| MR47 | 7.51 | 443.09897 | C22H19O10 | 1.354 | 267.06638(100),249.05563(25) | Glucuronidated methylchrysophanol | + |  |
| MR48 | 7.58 | 311.05548 | C17H11O6 | -2.03 | 311.05637(100),252.04189(11),223.04030(14) |  | + |  |
| MR49 | 7.60 | 303.05136 | C15H11O7 | 1.102 | 259.06137(100),241.05037(11),229.05038(12) | Taxifolin | + |  |
| H138 | 7.64 | 415.10376 | C21H19O9 | 0.734 | 277.05151(10),253.05128(100) | Chrysophanol 8-O-glucoside | + | + |
| MR50 | 7.69 | 473.07236 | C22H17O12 | -0.4 | 297.04007(5),253.05026(100) |  | + |  |
| MR51 | 7.69 | 355.14044 | C17H23O8 | 3.231 | 179.10689(71),164.08350(100),113.02306(46) |  | + |  |
| MP27 | 7.80 | 333.00729 | C15H9O7S | 11.5 | 253.05011(100) |  | + | + |
| MP28 | 7.83 | 421.11392 | C20H21O10 | -0.238 | 245.08192(100),230.05815(30) |  | + | + |
| H146 | 7.83 | 487.08823 | C23H19O12 | 0.063 | 311.05661(100) | Acetyl glucosylrhein | + |  |
| MR52 | 7.83 | 245.08107 | C14H13O4 | -3.518 | 245.08192(80),230.05826(100) |  | + |  |
| MP29 | 7.88 | 445.07724 | C21H17O11 | -0.886 | 269.04587(100),240.04268(1) | Glucuronidated emodin | + | + |
| MP30 | 7.89 | 429.08221 | C21H17O10 | -1.188 | 253.05017(100) | Glucuronidated chrysophanol | + | + |
| MR53 | 7.92 | 461.07233 | C21H17O12 | -0.475 | 285.04089(100) |  | + |  |
| MP31 | 7.93 | 475.08804 | C22H19O12 | -0.335 | 299.05646(91),284.03290(100) | Glucuronidated oxidized physcion | + | + |
| MP32 | 8.04 | 275.05646 | C14H11O6 | 1.268 | 231.06610(100),203.07083(17) |  | + | + |
| MP33 | 8.05 | 445.07678 | C21H17O11 | -1.920 |  |  | + | + |
| MP34 | 8.09 | 429.08240 | C21H17O10 | -0.746 | 253.05032(100) | Glucuronidated chrysophanol | + | + |
| H154 | 8.15 | 401.12451 | C21H21O8 | 0.796 | 281.08279(100),253.08733(3),239.07159(3) |  | + | + |
| MP35 | 8.16 | 364.99704 | C15H9O9S | -0.646 | 285.04028(100) | Sulfated oxidized aloe emodin | + | + |
| MR54 | 8.16 | 475.08804 | C22H19O12 | -0.335 | 299.05649(82),284.03299(100) | Glucuronidated oxidized physcion | + |  |
| H155 | 8.22 | 299.05624 | C16H11O6 | 0.43 | 299.05582(34),255.06624(100),231.06618(21) |  | + |  |
| H157 | 8.25 | 209.11781 | C12H17O3 | -2.428 | 209.11835(93),191.10747(100),165.12794(35),135.08067(45) |  | + |  |
| MR55 | 8.26 | 487.08832 | C23H19O12 | 0.248 | 311.05652(100) |  | + |  |
| MP36 | 8.29 | 461.07269 | C21H17O12 | 0.306 | 285.03970(100),240.00826(9) |  | + | + |
| MP37 | 8.31 | 349.00220 | C15H9O8S | -0.462 | 269.04538(100),240.04248(3) | Sulfated aloe emodin | + | + |
| H156 | 8.31 | 217.05025 | C12H9O4 | -1.760 | 217.05020(100),191.03421(35),175.03934(31) | Dimethyl-hydroxy-acetyl-chromene or isomers | + |  |
| H160 | 8.34 | 271.06137 | C15H11O5 | 0.639 | 271.06235(32),177.01918(11),151.00301(100),119.04925(57) | Naringenin | + |  |
| MP38 | 8.38 | 275.05627 | C14H11O6 | 0.577 | 275.05667(5),260.03281(100),232.03764(25) |  | + | + |
| MR56 | 8.41 | 311.05588 | C17H11O6 | -0.744 | 311.05664(100),223.08813(8) |  | + |  |
| MR57 | 8.49 | 321.20703 | C19H29O4 | -0.320 | 321.20746(100),303.19757(3),275.20227(13) |  | + |  |
| MR58 | 8.54 | 283.06049 | C16H11O5 | -2.497 | 283.0607(100),268.03763(31),265.05124(36),240.04228(83),237.05490(29) |  | + |  |
| MR59 | 8.55 | 459.09323 | C22H19O11 | -0.119 | 283.06158(100),240.04271(18) | Glucuronidated physcion | + |  |
| MP39 | 8.59 | 445.07755 | C21H17O11 | -0.190 | 269.04584(100),225.05553(1) | Glucuronidated emodin | + | + |
| MR60 | 8.62 | 303.05139 | C15H11O7 | 1.201 | 259.06119(27),231.0661(21),189.01871(100) | Dihydromorin | + |  |
| MR61 | 8.66 | 435.12857 | C21H23O10 | 2.091 | 259.09775(100),244.07439(27) |  | + |  |
| H168 | 8.68 | 313.07184 | C17H13O6 | 0.251 | 254.05836(100),253.05089(64) | Laccaic acid | + | + |
| MR62 | 8.74 | 269.04520 | C15H9O5 | -1.289 | 269.04581(100),241.05052(2),225.05571(5) |  | + |  |
| MR63 | 8.75 | 364.99783 | C15H9O9S | 1.518 | 283.02481(100),239.03383(28) | Sulfated oxidized aloe emodin | + |  |
| MR64 | 8.75 | 627.15283 | C34H27O12 | 3.238 | 269.08255(15),254.05806(100),253.05072(89) |  | + |  |
| MR65 | 8.77 | 459.09332 | C22H19O11 | 0.077 | 283.06152(100),240.04257(16) | Glucuronidated physcion | + |  |
| MP40 | 8.81 | 362.98163 | C15H7O9S | 0.012 | 283.02493(100),239.03452(31) | Sulfated rhein | + | + |
| MR66 | 8.96 | 283.06116 | C16H11O5 | -0.130 | 283.06119(4),240.07437(19),239.07127(100) |  | + |  |
| MR67 | 9.00 | 461.07269 | C21H17O12 | 0.306 | 285.04050(100),131.08380(9) | Glucuronidated oxidized emodin | + |  |
| MR68 | 9.03 | 365.23312 | C21H33O5 | -0.623 | 365.23364(100),321.24380(7),289.21768(3) |  | + |  |
| MP41 | 9.05 | 349.00220 | C15H9O8S | -0.462 | 269.04544(100) | Sulfated emodin | + | + |
| MP42 | 9.08 | 380.99240 | C15H9O10S | 0.550 | 301.03461(100) | Sulfated quercetin | + | + |
| MP43 | 9.19 | 364.99710 | C15H9O9S | -0.482 | 285.03998(100) | Sulfated oxidized emodin | + | + |
| MR69 | 9.25 | 437.14615 | C21H25O10 | 1.898 | 261.11346(100),235.13374(58),113.02323(47) |  | + |  |
| MP44 | 9.26 | 313.03531 | C16H9O7 | -0.210 | 299.01544(18),298.01221(100),270.01736(17) |  | + | + |
| MR70 | 9.26 | 291.12415 | C16H19O5 | 1.213 | 291.12427(9),247.13402(70),203.14365(100),134.07249(12) |  | + |  |
| H180 | 9.28 | 299.01993 | C15H7O7 | 0.683 | 299.02078(41),284.03281(9),255.02989(100),227.03505(11) | Laccaic acid D or isomers | + |  |
| MR71 | 9.41 | 453.28574 | C25H41O7 | -0.081 | 425.29178(26),407.28094(100) |  | + |  |
| MR72 | 9.46 | 343.08209 | C18H15O7 | -0.688 | 283.06152(100),269.0459(86) |  | + |  |
| MR73 | 9.47 | 283.06094 | C16H11O5 | -0.907 | 283.06165(100),269.04565(30),240.04269(89),195.13846(48) |  | + |  |
| H184* | 9.51 | 269.04532 | C15H9O5 | -0.842 | 269.04504(5),225.05515(100) | Aloe emodin | + |  |
| MP45 | 9.52 | 362.98160 | C15H7O9S | -0.071 | 283.02426(100),239.03400(42) | Sulfated rhein | + | + |
| MR74 | 9.59 | 301.03540 | C15H9O7 | 0.080 | 189.01822(100) |  | + |  |
| MR75 | 9.64 | 291.12424 | C16H19O5 | 1.522 | 291.12451(34),247.13379(55),203.14365(100) |  | + |  |
| MR76 | 9.76 | 269.04538 | C15H9O5 | -0.619 | 269.04666(1),225.05545(100) |  | + |  |
| MR77 | 9.79 | 378.97644 | C15H7O10S | -0.265 | 299.0202(100),255.02914(24) |  | + |  |
| MR78 | 9.91 | 349.23813 | C21H33O4 | -0.867 | 349.23883(100),303.23315(20) |  | + |  |
| MP46 | 9.92 | 392.99246 | C16H9O10S | 0.686 | 313.03491(48),298.01230(100) | Sulfated-dioxidized acetyl dithranol | + | + |
| H196 | 9.93 | 287.05634 | C15H11O6 | 0.797 | 243.06683(100),225.05597(7),163.00316(23),123.04427(23) | 3,5-Dihydroxy-2-[2-(4-hydroxyphenyl) acetyl]benzoic acid | + |  |
| MR79 | 10.14 | 311.05615 | C17H11O6 | 0.124 | 268.06946(8),267.06656(81),225.05272(12),224.04762(100) |  | + |  |
| MR80 | 10.31 | 386.17957 | C22H28O3NS | 0.084 | 386.18048(6),349.23907(2) |  | + |  |
| MP47 | 10.39 | 349.00201 | C15H9O8S | -1.006 | 269.04541(100) | Sulfated emodin | + | + |
| MR81 | 10.42 | 376.99704 | C16H9O9S | -0.625 | 297.04037(100),255.06535(23) | Sulfated-oxidized acetyl dithranol | + |  |
| MP48 | 10.46 | 313.03543 | C16H9O7 | 0.173 | 298.01071(45),254.02205(48),226.02448(23) |  | + | + |
| H202 | 10.53 | 299.05630 | C16H11O6 | 0.631 | 299.05627(72),255.06615(55),240.04266(100) | Oxidized physcion | + |  |
| H204 | 10.69 | 303.08786 | C16H15O6 | 1.480 | 303.08804(5),259.09778(56),244.07423(100) | Methylcatechin | + |  |
| MR82 | 10.69 | 386.17957 | C22H28O3NS | 0.084 | 349.23907(100) |  | + |  |
| MP49 | 10.74 | 333.00729 | C15H9O7S | -0.470 | 253.05078(100) | Sulfated chrysophanol | + | + |
| MR83 | 10.77 | 349.23816 | C21H33O4 | -0.781 | 349.23877(100),331.22772(3),305.24905(16) |  | + |  |
| H206 | 10.78 | 299.01993 | C15H7O7 | 0.683 | 299.02011(100),284.03287(7),255.02975(43),240.04346(21) | Laccaic acid D or isomers | + | + |
| MR84 | 10.90 | 378.97653 | C15H7O10S | -0.027 | 299.01974(100) |  | + |  |
| MR85 | 11.00 | 163.07544 | C10H11O2 | -6.211 | 147.04414(100) | Frambinone | + |  |
| MP50 | 11.05 | 333.00732 | C15H9O7S | -0.380 | 253.05078(100) | Sulfated chrysophanol | + | + |
| MP51 | 11.24 | 363.01813 | C16H11O8S | 0.327 |  | Sulfated physcion | + | + |
| H209 | 11.31 | 396.96558 | C18H5O9S | -0.997 | 353.97870(13),352.97723(47),295.02563(23),116.92754(100) |  | + | + |
| MP52 | 11.54 | 285.04105 | C15H9O6 | 2.065 | 285.03891(21),259.05084(26),258.04855(61),241.04779(38),240.04034(100) | Oxidized aloe emodin | + | + |
| H211* | 11.66 | 283.02466 | C15H7O6 | -0.534 | 283.02515(15),257.04575(100),240.03896(14),239.03493(77),183.04445(21) | Rhein | + | + |
| MP53 | 11.90 | 364.99692 | C15H9O9S | -0.975 |  |  | + | + |
| MP54 | 12.10 | 349.00217 | C15H9O8S | -0.548 | 269.04541(100) |  | + | + |
| MP55 | 12.17 | 363.01816 | C16H11O8S | 0.410 | 283.06119(100) | Sulfated physcion | + | + |
| MR86 | 12.22 | 349.23813 | C21H33O4 | -0.867 | 349.23889(100),331.22760(1),303.23337(36) |  | + |  |
| MR87 | 12.29 | 261.11301 | C15H17O4 | -0.851 | 261.11359(31),235.13388(100) |  | + |  |
| MR88 | 12.43 | 313.03540 | C16H9O7 | 0.077 | 269.04529(37),183.13809(100),129.09065(62) |  | + |  |
| H214 | 12.91 | 299.02005 | C15H7O7 | 1.084 | 299.01859(16),273.04022(84),255.02971(100),227.03375(26) | Laccaic acid D or isomers | + | + |
| MP56 | 12.94 | 301.03537 | C15H9O7 | -0.019 |  |  | + | + |
| MP57 | 12.95 | 364.99704 | C15H9O9S | -0.646 | 285.03964(100) | Sulfated oxidized emodin | + | + |
| MP58 | 13.10 | 313.03525 | C16H9O7 | -0.402 | 287.0556(24),269.04535(7),254.02161(100) |  | + | + |
| H215* | 13.23 | 269.04532 | C15H9O5 | -0.842 | 269.04605(100),225.05598(6) | Emodin | + | + |
| MR89 | 13.30 | 279.16043 | C16H23O4 | 0.887 | 235.16982(9),191.17987(91) |  | + |  |
| MP59 | 13.30 | 299.05594 | C16H11O6 | -0.573 | 273.04111(33),255.02930(100) | Hydroxyl-methyl-aloe emodin | + | + |
| MP60 | 13.52 | 349.00229 | C15H9O8S | -0.204 | 269.04526(100) | Sulfated emodin | + | + |
| MP61 | 13.68 | 364.99707 | C15H9O9S | -0.564 | 285.04041(100) | Sulfated oxidized aloe emodin | + | + |
| H217* | 14.67 | 253.05023 | C15H9O4 | -1.589 | 253.05133(100),225.05586(6) | Chrysophanol | + |  |
| H216 | 14.69 | 255.06746 | C15H11O4 | 4.618 | 255.06502(100),213.05533(12),151.00247(8) | Pterostilbene or isomers | + |  |
| H218 | 15.07 | 387.12427 | C24H19O5 | 1.222 | 240.07521(10),239.07184(100) |  | + |  |
| H220* | 16.28 | 283.06198 | C16H11O5 | 2.767 | 283.06216(98),268.03836(8),240.04330(100) | Physcion | + |  |
| H223 | 19.60 | 507.10889 | C30H19O8 | 0.689 | 489.09738(7),279.23297(36),253.05072(100) | Rheidin B | + | + |

*Compounds validated by reference substances
